# Genome sequencing and comparative analysis of *Wolbachia* strain *w*AlbA reveals *Wolbachia*-associated plasmids are common

**DOI:** 10.1371/journal.pgen.1010406

**Published:** 2022-09-19

**Authors:** Julien Martinez, Thomas H. Ant, Shivan M. Murdochy, Lily Tong, Ana da Silva Filipe, Steven P. Sinkins

**Affiliations:** MRC-University of Glasgow Centre for Virus Research, Glasgow, United Kingdom; University of Warwick, UNITED KINGDOM

## Abstract

*Wolbachia* are widespread maternally-transmitted bacteria of arthropods that often spread by manipulating their host’s reproduction through cytoplasmic incompatibility (CI). Their invasive potential is currently being harnessed in field trials aiming to control mosquito-borne diseases. *Wolbachia* genomes commonly harbour prophage regions encoding the *cif* genes which confer their ability to induce CI. Recently, a plasmid-like element was discovered in *w*Pip, a *Wolbachia* strain infecting *Culex* mosquitoes; however, it is unclear how common such extra-chromosomal elements are in *Wolbachia*. Here we sequenced the complete genome of *w*AlbA, a strain of the symbiont found in *Aedes albopictus*, after eliminating the co-infecting and higher density *w*AlbB strain that previously made sequencing of *w*AlbA challenging. We show that *w*AlbA is associated with two new plasmids and identified additional *Wolbachia* plasmids and related chromosomal islands in over 20% of publicly available *Wolbachia* genome datasets. These plasmids encode a variety of accessory genes, including several phage-like DNA packaging genes as well as genes potentially contributing to host-symbiont interactions. In particular, we recovered divergent homologues of the *cif* genes in both *Wolbachia-* and *Rickettsia*-associated plasmids. Our results indicate that plasmids are common in *Wolbachia* and raise fundamental questions around their role in symbiosis. In addition, our comparative analysis provides useful information for the future development of genetic tools to manipulate and study *Wolbachia* symbionts.

## Author summary

*Wolbachia* is the most common bacterial symbiont of arthropods, being found in about half of terrestrial species around the globe. It is transmitted from mother to offspring, can spread rapidly by inducing various forms of reproductive parasitism and often provides protection against viral pathogens. These properties are being harnessed by disease control interventions introducing *Wolbachia* into wild populations of virus-transmitting mosquitoes. Here we sequenced the genome of *w*AlbA, a strain of the symbiont naturally found in *Aedes albopictus* mosquitoes and demonstrate that it is associated with two extrachromosomal plasmid elements. We then reanalysed publicly available sequencing data and found that plasmids are much more common in *Wolbachia* than previously thought. Some of them carry genes potentially important for symbiosis and reproductive manipulation as well as phage-like genes that may allow them to move between symbiont strains. Our findings provide a new framework for studying *Wolbachia* and will help the future development of genetic tools for manipulating symbiont genomes.

## Introduction

*Wolbachia* is the most abundant heritable bacterium in arthropods, present in around half of the species worldwide [1], as well as being an obligate symbiont of filarial nematodes [2]. It is primarily inherited through the female germline and has evolved various ways to spread through host populations by manipulating arthropod reproduction. Reproductive alterations include several forms of sex-ratio distortions and cytoplasmic incompatibility (CI), a type of selective sterility providing a reproductive advantage to female hosts carrying the symbiont [3]. Many *Wolbachia* strains are also capable of inhibiting viral replication [4–7] and this phenotype, combined with the self-spreading mechanism of CI, allowed the development of novel strategies for controlling mosquito-borne diseases [8–10].

*Wolbachia* is found exclusively within the host cell environment, and this has hampered the use of genetic tools to manipulate and study its genome at the mechanistic level. Nevertheless, genome research has led to considerable progress in understanding *Wolbachia* biology [11–15]. *Wolbachia* is commonly associated with prophage WO, a temperate bacteriophage integrated as a prophage region into the symbiont’s chromosome. Prophage WO has an important role in *Wolbachia*‘s evolution by allowing the transfer of genetic material between symbiont genomes [16]. Prophage WO also carry an accessory region called the Eukaryotic Association Module (EAM) which encodes a variety of genes that are eukaryotic-like in length, origin and/or predicted function such as ankyrin domain-containing proteins. Moreover, the EAM often carries the two syntenic genes *cifA* and *cifB* responsible for the CI phenotype [17–19]. While the mobility of phage WO has contributed to horizontal gene transfer over long evolutionary periods, evidence of active phage replication and production of virus particles remain limited to a few examples [19–22]. In addition, many prophage regions display signs of degradation through pseudogenization of core phage genes and chromosomal rearrangements [16].

Recently, a mobile genetic element was discovered in *w*Pip, a strain of *Wolbachia* infecting *Culex pipiens* mosquitoes [23]. The 9,228 bp extrachromosomal and circular element bears the hallmarks of a candidate plasmid and was therefore named pWCP for “plasmid of *Wolbachia* endosymbiont in *C*. *pipiens*”. Since this discovery, no other *Wolbachia*-associated plasmids has been described and it is unclear how common these elements are and whether they play an important role in *Wolbachia*-host interactions.

There is abundant literature highlighting the contribution of plasmids in the horizontal transfer of adaptive traits such as antibiotic resistance and virulence in free-living bacteria [24–26]. Plasmids have also been described in arthropod symbionts and they often carry genes potentially involved in symbiosis. For instance, ankyrin repeat-containing and toxin-like genes are commonly found in the plasmids of *Cardinium* [27,28], *Spiroplasma* [29,30] and *Rickettsia* symbionts [31]. In *Spiroplasma* strain MSRO infecting *Drosophila melanogaster*, a male-killing phenotype is mediated by the *spaid* toxin encoded on plasmid pSMSRO [32]. Homologues of the *Wolbachia cif* genes have also been identified on plasmid pLbaR in *Rickettsia felis*; the presence of *R*. *felis* in *Liposcelis bostrichophila* correlates with parthenogenesis [33,34].

The invasive ‘Asian tiger’ mosquito *Aedes albopictus*, a vector of arboviruses such as dengue, chikungunya and Zika, harbours two co-infecting strains of *Wolbachia* called *w*AlbA and *w*AlbB. *w*AlbA has a lower overall density and in females is largely restricted to the ovaries, while *w*AlbB has a somewhat wider tissue distribution [35,36], a pattern that is also observed in other doubly-infected systems [37,38]. Following transfer into the naturally *Wolbachia*-free *Ae*. *aegypti*, much wider tissue distribution occurs for both strains in this novel host, but their relative density was reversed; however, despite its lower density *w*AlbB was a much more efficient inhibitor of arbovirus transmission in *Ae*. *aegypti* than *w*AlbA [6]. Subsequently *w*AlbB has been deployed in Malaysia for dengue control [8]. Several genomes of *w*AlbB have been sequenced [15,39,40], but due to the technical difficulties associated with its lower density and co-presence of *w*AlbB, no *w*AlbA genome has been reported to date.

Here we report the complete genome of *Wolbachia* strain *w*AlbA and two new associated plasmids, together with a number of similar plasmids and chromosomal islands in existing *Wolbachia* genome assemblies.

## Results

### *w*AlbA harbours prophage regions and multiple pairs of *cif* genes

In order to sequence the *w*AlbA genome, we generated a singly-infected *Ae*. *albopictus* line by eliminating the co-infecting strain *w*AlbB (see methods). We found that *w*AlbA is most abundant in the ovaries and its tissue distribution is not affected by the presence of *w*AlbB (S1A Fig). In addition, *w*AlbA does not affect the fecundity of female mosquitoes and is able to induce CI (S1B and S1C Fig). However, *w*AlbA is unable to rescue CI in crosses with doubly-infected males, suggesting that *w*AlbA and *w*AlbB carry incompatible pairs of *cif* genes (S1C Fig).

**Fig 1:**
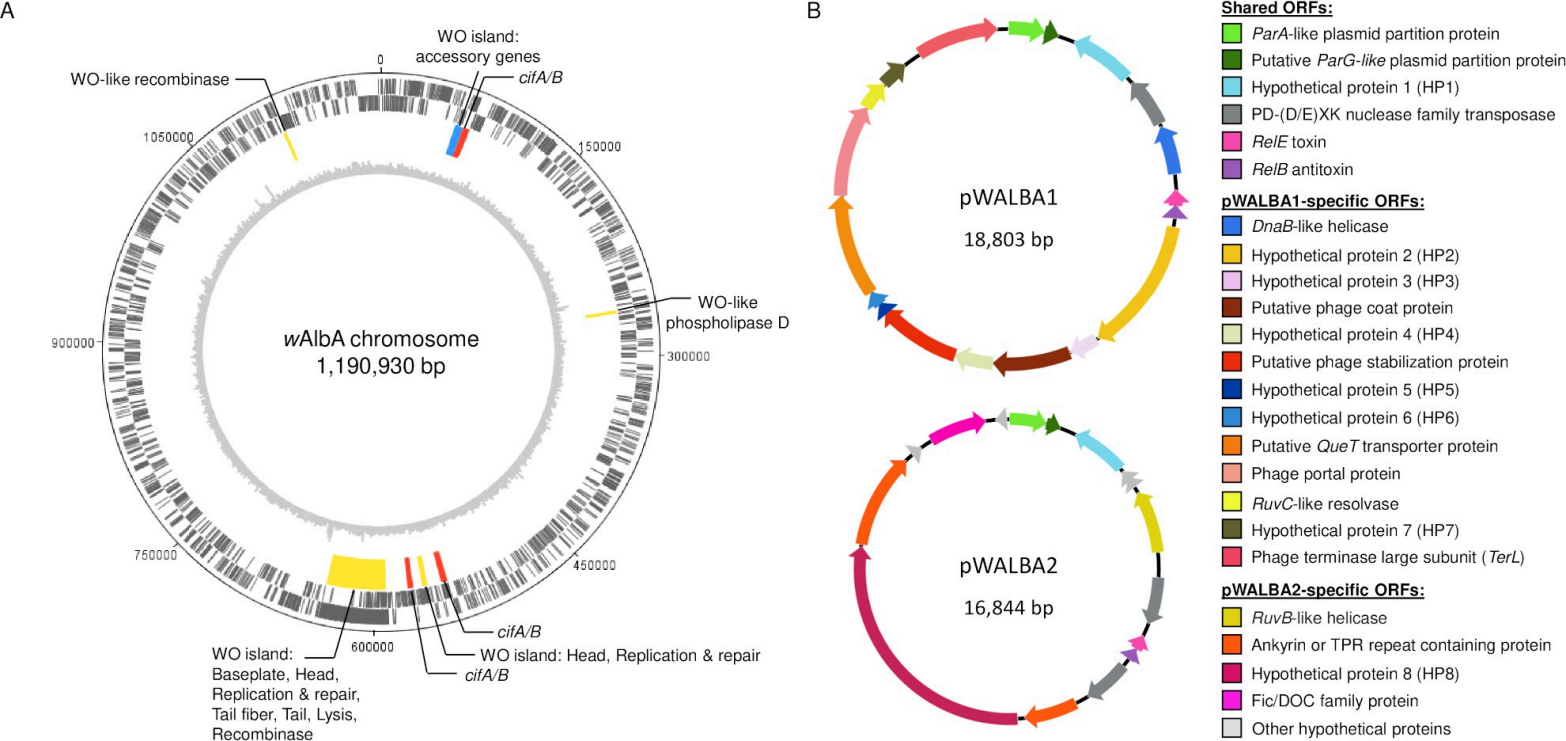
Genome map of the *w*AlbA chromosome and plasmids. (A) *w*AlbA chromosome. Two outer circles: protein-coding genes on forward and reverse strands (black). Third circle: WO prophage regions (yellow: phage core genes, red & blue: phage eukaryotic modules). Inner grey circle: Illumina sequencing depth per 2,000 bp window from a non-WGA mosquito sample (see methods). (B) *w*AlbA plasmids and predicted coding sequences.

**Fig 2:**
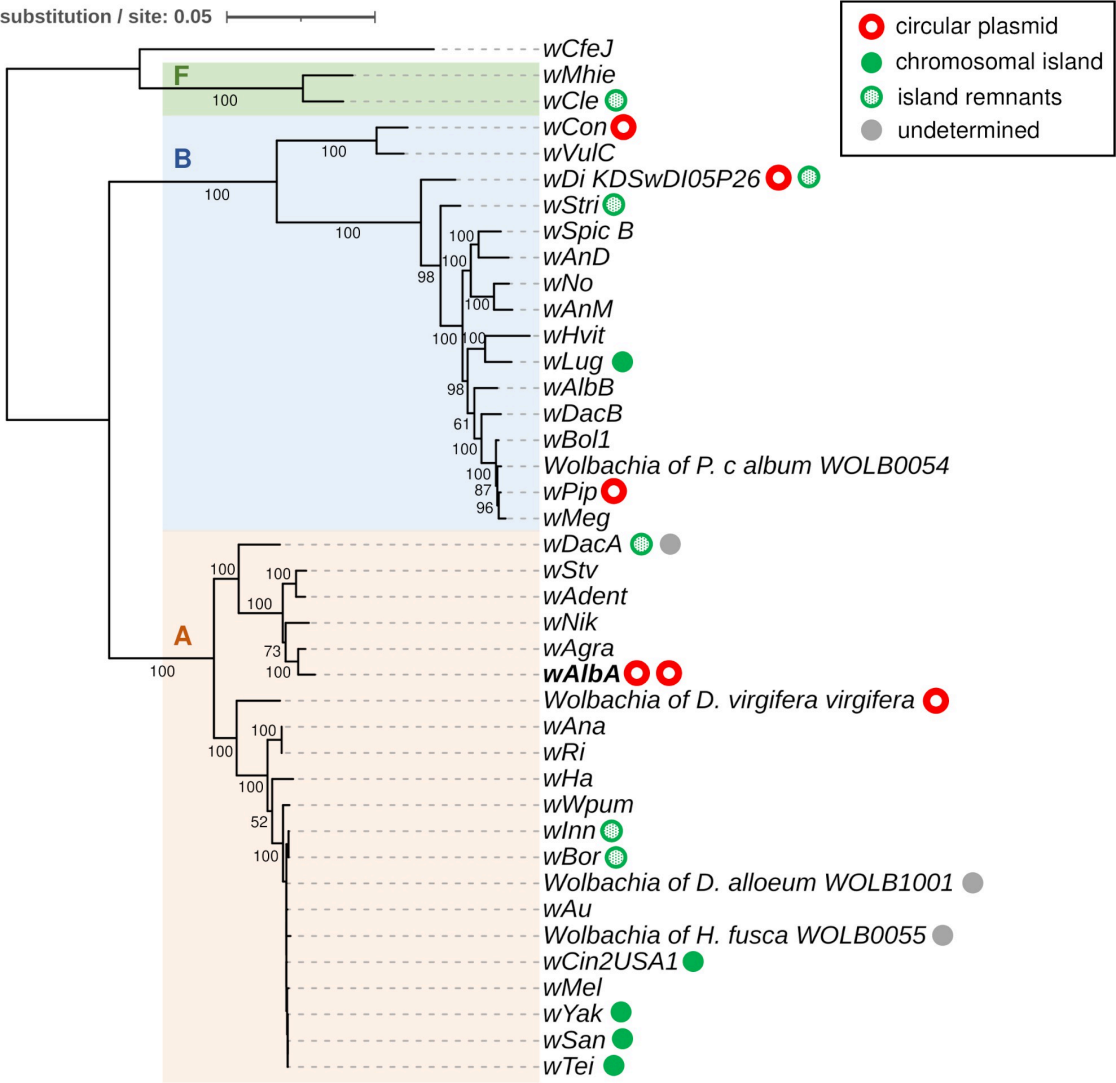
*Wolbachia* strain phylogeny and distribution of plasmid-like elements. Maximum Likelihood phylogeny based on the concatenated nucleotide alignment of 36 *Wolbachia* core genes using the GTR GAMMA substitution model. Branch support was assessed with 1,000 bootstrap replicates. Capital letters indicate *Wolbachia* supergroups. Circles indicate strains in which plasmid-like elements were identified.

We assembled a complete 1,190,930 bp *Wolbachia* genome from *w*AlbA-infected ovaries (circularity determined by the Unicycler pipeline, see methods, Fig 1A) which is similar in size and gene number to other complete *Wolbachia* genomes (S1 Table). *w*AlbA belongs to the arthropod-specific *Wolbachia* supergroup A (Fig 2) and harbours a large WO prophage region (47,475 bp, Fig 1A) comprising a complete set of structural and non-structural gene modules thought to be essential for the production of phage particles (S2 Table). However, this region displays a sequencing depth similar to the rest of the chromosome, suggesting no active replication of WO phage (Fig 1A). We also found smaller WO phage islands containing core phage genes, often with signs of pseudogenization, and accessory genes commonly found in the phage eukaryotic association module (Fig 1A and S2 Table). This includes three nearly identical pairs of the cytoplasmic incompatibility genes *cifA* and *cifB*, all harbouring a *cifB* deubiquitinase domain that is characteristic of Type I *cif* homologues [33].

### *w*AlbA is associated with two plasmid-like elements

In addition to the *w*AlbA genome, we assembled two circular extrachromosomal elements, 18,803 and 16,844 bp in size, that show strong similarities to pWCP, the *Wolbachia*-associated plasmid found in *C*. *pipiens* (Fig 1B). Based on the comparative analysis below, we named these plasmids pWALBA1 and pWALBA2 (for plasmids of *Wolbachia* endosymbiont *w*AlbA 1 and 2). Sequencing depth was evenly distributed along the two plasmid genomes with only three low-frequency single nucleotide polymorphisms detected, indicating near complete plasmid clonality within our *w*AlbA-infected mosquito line (S2 Fig). Using specific PCR primers, we confirmed the association of the two plasmids with *w*AlbA, whereas no amplification was observed in the *Ae*. *albopictus* Aa23 cell line infected with *w*AlbB only (S3 Fig). pWALBA1 showed a higher copy number per *Wolbachia* cell than pWALBA2, although pWALBA2 copies tended to increase in older female mosquitoes (S4A Fig). Importantly, the relative amounts of the two plasmids per mosquito were strongly correlated with *w*AlbA density, further supporting their association with the symbiont strain (S4B Fig).

Similar to pWCP, both *w*AlbA plasmids encode a *ParA*-like plasmid partitioning gene (HHpred probability > 99%), a hypothetical protein with strong similarity to a *ParG*-like plasmid partition gene and other DNA-binding proteins found on extra-chromosomal elements (e.g. phage transcriptional *Arc* repressor, HHpred p > 99%), a hypothetical protein (HP1), a *RelB/E* toxin-antitoxin addiction module (HHpred p > 98%) as well as one or two transposases (Fig 1B, S3 Table). pWALBA1 shares additional genes with pWCP, namely a *DnaB*-like helicase (HHpred p = 99.96%) and a *TerL*-like phage terminase large subunit (HHpred p = 100%), but contrary to pWCP, these two genes do not show sign of pseudogenization. pWALBA1 encodes other phage-like proteins, namely a portal protein (HHpred p = 100%), a putative phage stabilization protein (HHpred p = 99.44%) and a hypothetical protein with weak homologies to a phage coat protein (HHpred p = 64.73%, S3 Table). Other pWALBA1 genes include a putative *QueT*-like queuosine transporter (HHpred p = 95.17%), a *RuvC*-like resolvase (HHpred p = 98.17%) and several hypothetical proteins absent in pWCP. We did not find phage-like genes in pWALBA2 but instead genes encoding a Fic/DOC family protein (HHpred p = 100%), a *RuvB*-like helicase (HHpred p = 99.02%) and other proteins with no predicted functions, some of which carry ankyrin or tetratricopeptide (TPR) repeat domains (Fig 1B and S3 Table).

### Plasmids and related chromosomal islands are widespread in *Wolbachia*

**Fig 3:**
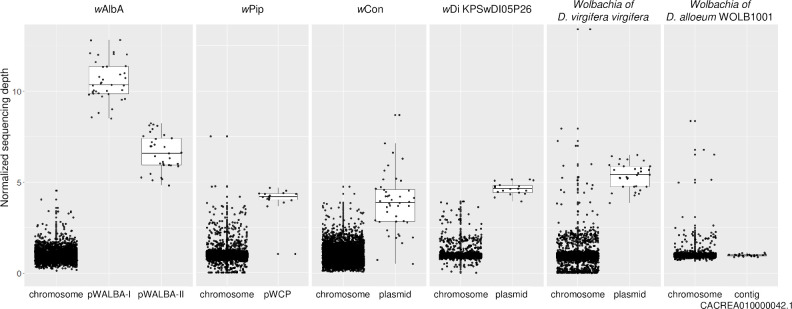
Relative sequencing depth of *Wolbachia*-associated plasmid-like elements. Each dot indicates the mean sequencing depth in 500 bp windows divided by the mean chromosome depth.

In order to investigate how common plasmid-like elements are in *Wolbachia*, we conducted TBLASTN searches of pWALBA1 and pWALBA2 coding sequences in publicly available *Wolbachia* genomes. We found plasmid-like regions with similar gene organization to *w*AlbA plasmids in 47 out of 189 *Wolbachia* assemblies (~20% of the strains, several assemblies for some strains) and none were found in strains from nematodes (S4 Table). Their distribution is patchy across the *Wolbachia* phylogeny, suggesting that plasmids are often horizontally-transferred between symbiont strains and tend to be lost over long evolutionary timeframes (Fig 2). However, it is possible that plasmid sequences are missing from some *Wolbachia* assemblies. Indeed, filtering out contigs from final assemblies based on a lack of homology with known *Wolbachia* sequences or due to large differences in sequencing depth is common practice. For instance, not all draft assemblies of the *Wolbachia* infecting *Diabrotica virigifera virgifera* contained plasmid-like sequences (S4 Table). Moreover, we found a plasmid-like contig in several isolates of strain *w*Di [41] that was absent in other published *w*Di assemblies [42]. However, by re-assembling the raw reads from the latter study, we retrieved the corresponding circular plasmid genome that had not been reported (isolate *w*Di_KPSwDI05P26). We circularized additional plasmid-like elements from the *w*Con strain (SRA accession: SRR7213553), from a *Wolbachia*-infected sample of the *Diabrotica virgifera virgifera* beetle (SRA accession: SRR1106544) and from a sample labelled *Insecta*_WOLB1166 (mixture of *Wolbachia*-infected insect species, SRA accession: SRR748268). All circular plasmids show higher sequencing depth relative to their respective *Wolbachia* genome, ranging from ~4 to 10x, suggesting that they are maintained as multiple copies within bacterial cells (Fig 3). In some cases, copy number could not be estimated due to raw sequencing data being unavailable (*w*DacA) or to the presence of several *Wolbachia* strains in the same sample as indicated by multiple peaks in the sequencing depth distribution (*Insecta*_WOLB1166, *Dactylopius coccus* WOLB1009 and *Megaselia abdita* WOLB1013).

**Fig 4:**
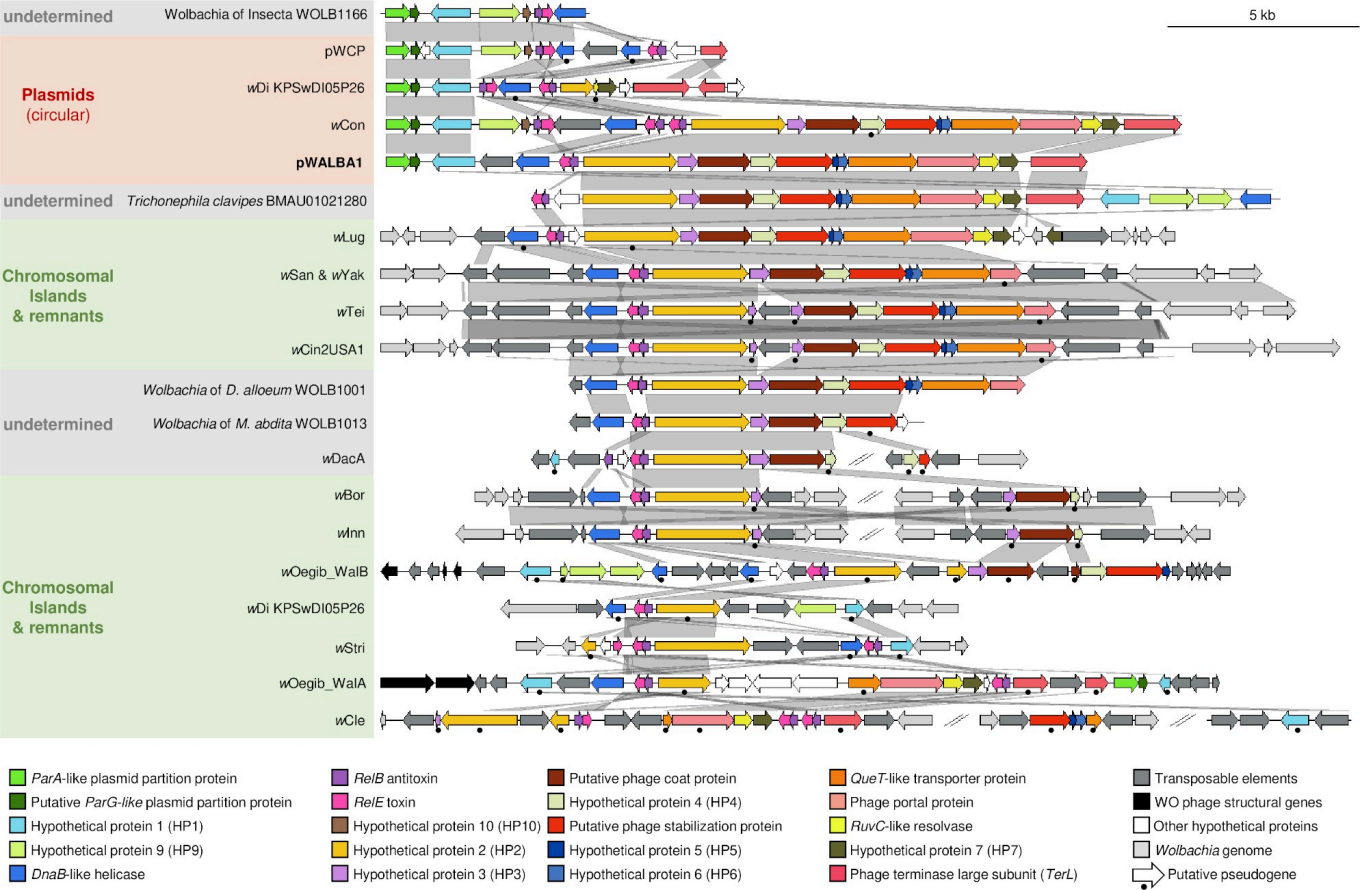
Gene organisation and comparison of pWALBA1-like sequences. Similarity is indicated by gene colours (TBLASTN) and by the grey areas between sequences (BLASTN) where darker grey means more similar.

Some linear contigs could not be circularized but show a similar sequencing depth relative to other *Wolbachia* contigs and could be single-copy plasmids or be part of the bacterial chromosome (e.g. in *Wolbachia* of *Diachasma alloeum* WOLB1001, Fig 3). In support of the latter hypothesis, we found several genomic regions similar to pWALBA1 integrated on the *Wolbachia* chromosome (Fig 4). These chromosomal islands include a twelve gene region previously named “Dozen island” present in *Wolbachia* strains from *Drosophila teissieri*, *santomea* and *yakuba* that encompasses the variable region of pWALBA1-like plasmid genomes [43]. In most cases, these islands are flanked by transposable elements and show varying degrees of chromosomal rearrangements and degradation with genes carrying premature stop codons or being truncated by transposase sequences (Fig 4). An interesting observation is that plasmid-like islands in strains *w*Oegib-WalA and *w*Oegib-WalB found in *O*. *gibbosus* spiders are located next to WO prophage regions (Fig 4).

**Fig 5:**
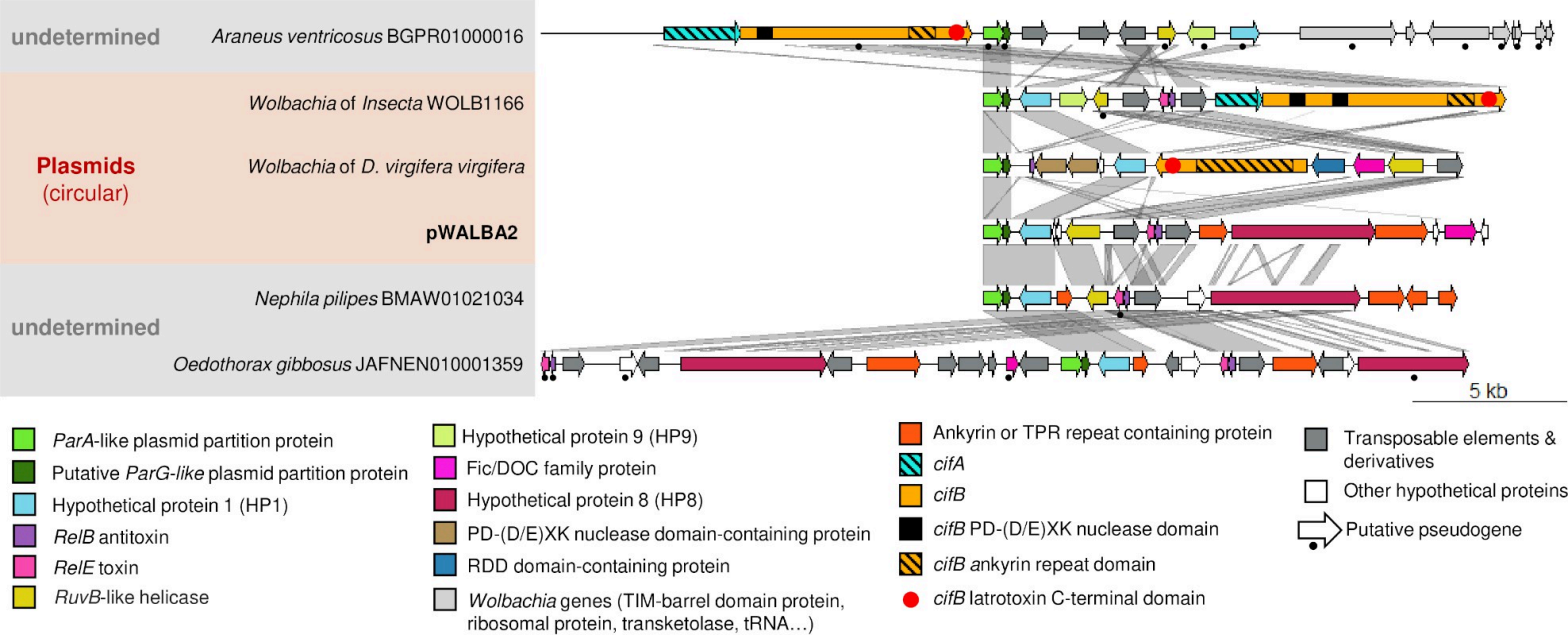
Gene organisation and comparison of other plasmid-like sequences. Similarity is indicated by gene colours (TBLASTN) and by the grey areas between sequences (BLASTN) where darker grey means more similar.

Other circular plasmid sequences, including pWALBA2, were found to share the same set of core genes with the plasmids described above (*ParA*-like, *ParG*-like, HP1, *RelB/E*) while encoding different genes in their accessory region (Fig 5). These include a Fic/DOC family protein and other hypothetical proteins carrying ankyrin, PD-(D/E)XK nuclease domains and RDD (repeated D domain). PD-(D/E)XK nucleases are a large and diverse family of proteins including restriction endonucleases, Holliday junction resolvases, transposases, and DNA repair enzymes [44]. The two plasmid-encoded nucleases in *D*. *virgifera virgifera* are unrelated to *cifB* genes which also carry PD-(D/E)XK domains, instead they are homologues of nucleases found in some WO phage head modules, the function of which is unknown [45]. In addition, the plasmid found in the *Insecta*_WOLB1166 sample encodes a *cifA/B* gene pair and the one from *D*. *virgifera virgifera* a *cifB* homologue lacking the 5’ end of the gene (Fig 5). In both cases, *cifB* harbours an ankyrin-repeat region as well as a C-terminal latrotoxin domain which was only found previously in divergent Type V *cifB* homologues (Fig 5) [33].

Plasmid-like sequences were also identified in draft genome assemblies of several species of spider (*Trichonephila clavata*, *T*. *clavipes*, *Araneus ventricosus*, *Nephila pilipes*, *Oedothorax gibbosus*) and one in the parasitic wasp *Cotesia glomerata* (S4 Table and Figs 4 and 5). Most of these sequences are made of a short contig, with no eukaryotic-like genes and could be *Wolbachia*-associated plasmids or chromosomal islands that were not filtered out from the host genome assembly. For instance, *O*. *gibbosus* is known to host two *Wolbachia* strains [46]. Nevertheless, two large spider contigs (*A*. *ventricosus* contig BGPR01000016.1, *T*. *clavipes* contig BMAU01021280.1) only showed homologies to *Wolbachia* in the region overlapping the plasmid-like sequences and could be cases of *Wolbachia*-to-spider horizontal transfers (S5 Fig). However, we observed large variation in sequencing depth in the regions flanking the two putative plasmid inserts and low numbers of long Nanopore reads supporting these insertions (S5 Fig). Therefore, we conclude that these are most likely chimeric contigs generated during the genome assembly of a *Wolbachia*-infected sample.

### Pervasive gene flow between *Wolbachia* and plasmid genomes

Phylogenetic analysis of the genes found in *Wolbachia* plasmids and chromosomal islands revealed the dynamic nature of lateral gene transfer and recombination between plasmids and the bacterial genome. Genes located on chromosomal islands do not form a monophyletic group which indicates that plasmid sequences have integrated into *Wolbachia* genomes more than once (S6–S9 Figs). The phylogenetic relationships of genes in the Dozen island found in the *w*Tei-*w*Yak-*w*San clade and other closely-related *Wolbachia* strains suggest, however, a single origin of this island that has most likely codiverged with the bacterial chromosome following its integration. Genes of the Dozen island are on average more similar to homologues found in pWALBA1, while genes located on other islands such as in *w*Lug and *w*Stri tend to be more closely-related to the *w*Con plasmid (S6–S8 Figs). The opposite pattern was found for the putative coat protein where the *w*Con homologue is more similar to its counterparts located within the Dozen island suggesting a possible recombination event. Another case of a probable recombination is observed between pWALBA1 and pWALBA2 since they carry divergent *ParA*-like plasmid partition genes while their genes encoding HP1 are closest relatives (S6 Fig).

Several plasmid genes also have closely-related homologues on the bacterial chromosome located outside of plasmid-like islands, although we cannot rule out that they are island remnants. In the case of *ParA*-like homologues, chromosomal versions of the gene form a separate clade and, interestingly, they are all fused with the downstream *ParG-like* gene, as opposed to plasmid-encoded homologues (S6 Fig). For other genes, there is evidence of multiple horizontal transfers between plasmids and *Wolbachia* genomes, including with WO prophage regions. RelB/E addiction modules are widespread in *Wolbachia* genomes [47] and their phylogenetic distribution does not suggest single origins for chromosomal, WO phage and plasmid homologues but rather multiple transfers between the different genomic entities (S10 Fig). Similarly, the two PD-(D/E)XK nucleases located on the *Wolbachia* plasmid in *D*. *virgifera virgifera* are related to different lineages of WO phage nucleases (S9 Fig). Finally, the plasmid Fic/DOC family proteins of pWALBA2 and *D*. *virgifera virgifera* group with two different clades of chromosomal Fic proteins commonly found in *Wolbachia* genomes, again suggesting multiple gene transfers between plasmids and *Wolbachia* genomes [47] (S9 Fig).

### Are plasmids vectors of *cif* genes across the *Rickettsiales*?

**Fig 6:**
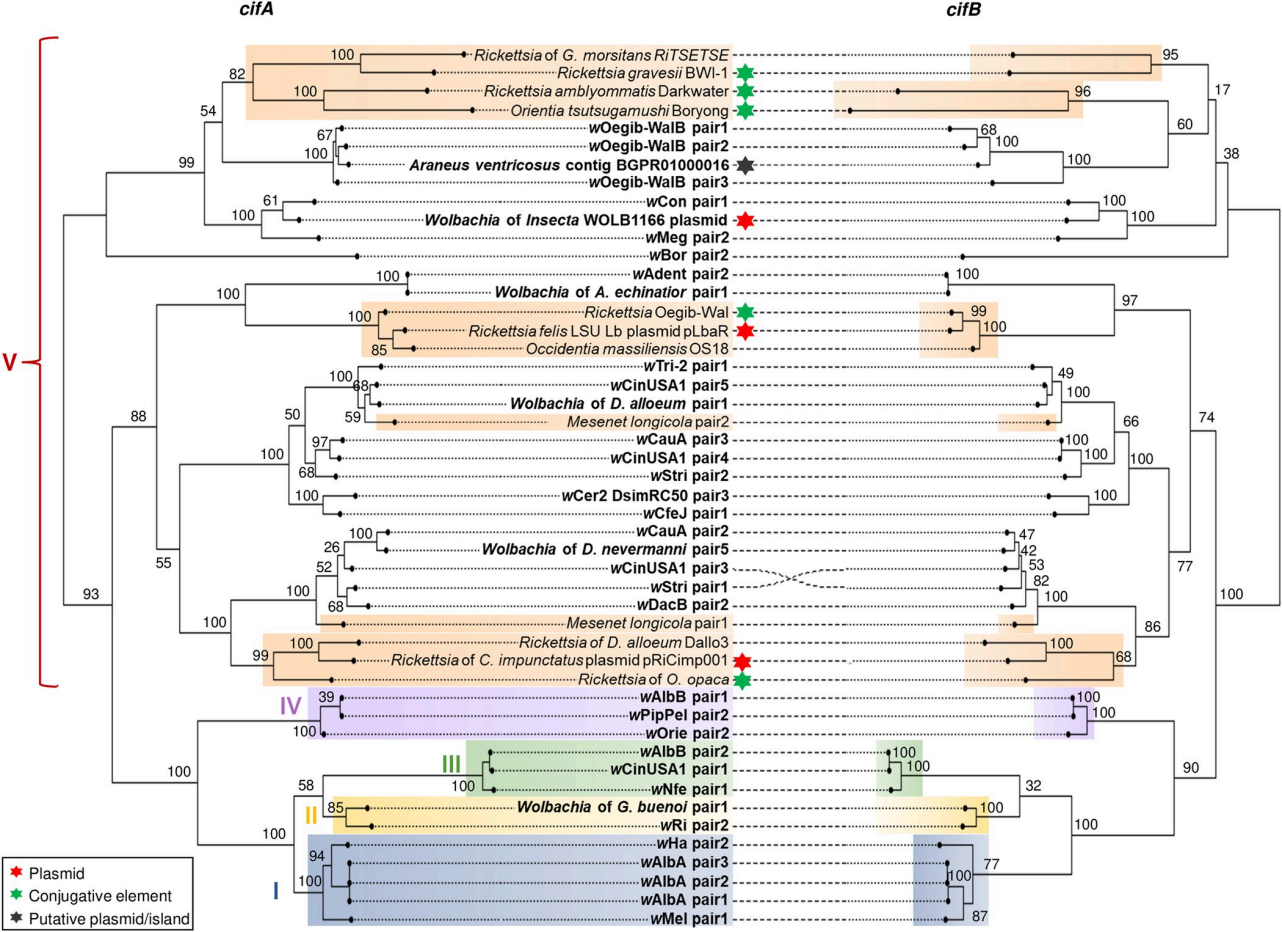
*Cif* gene phylogeny. Maximum Likelihood phylogeny based on the nucleotide alignment of *cifA* (left) and their cognate *cifB* genes (right) using the GTR GAMMA substitution model. The tree is midpoint rooted. Previously defined *cif* Types I-IV are labelled and colour-shaded (blue/yellow/green/purple). Orange shading indicate Type V-like homologues found outside *Wolbachia* symbionts. *Wolbachia* homologue labels are in bold. Bootstrap values were estimated from 100 replicates.

The *cif* genes are most of the time associated with WO prophage regions in *Wolbachia*. Previous studies have delimited multiple *cif* clades denominated as Type I to IV and a proposed Type V comprising homologues carrying additional domains on the C-terminal part of the cifB protein. *cif* homologues have also been reported in other *Rickettsiales*, some of them carried by plasmids [33,34,48,49]. We investigated the evolutionary origin of the *cif* genes located on *Wolbachia*-associated plasmid sequences and found that they group with Type V homologues (Fig 6), which is consistent with the length and predicted domains of their cifB protein (Fig 5). Unlike Type I-IV homologues, Type V *cif* genes are composed of several divergent clades with both *Wolbachia* and non-*Wolbachia* homologues and may not form a single monophyletic group (Fig 6). While the exact position of the tree root is currently unknown, we found evidence for several horizontal transfers of Type V *cif* genes between *Wolbachia* and other arthropod symbionts (Fig 6). Interestingly, the *cif* genes found in *Wolbachia*- and *Rickettsia*-associated plasmids do not cluster together suggesting independent acquisitions of *cif* genes by plasmids. In addition, we often found genes of the conjugation machinery in the regions flanking non-*Wolbachia cif* genes. In the chromosome of *Orientia tsutsugamushi* and the *Rickettsia* symbiont Oegib-Wal of the spider *O*. *gibbosus*, the *cif* genes are located next to an Integrative Conjugal Element (ICE) named *Rickettsiales* amplified genetic element (RAGE), a type of mobile genetic element commonly found in *Rickettsia* genomes and plasmids [31,50] (Figs 6 and S11). Other non-*Wolbachia cif* genes were located on short contigs but we often found genes of the conjugative machinery in the flanking regions suggesting that they are also associated with a chromosomal ICE or a conjugative plasmid (Fig 6).

## Discussion

Until the recent discovery of the pWCP element [23], it was assumed that plasmids were either absent or extremely rare in *Wolbachia*. Baião et al. (2021) later speculated that a putative plasmid was present in *w*Con and that the Dozen island might stem from an integrated plasmid. Our study shows that these elements have been largely overlooked, likely as a consequence of them being discarded as contaminants or assumed to be part of the bacterial chromosome in *Wolbachia* sequencing projects. Our characterization of plasmids associated with *w*AlbA and other *Wolbachia* strains will facilitate the identification of related elements in future studies and should encourage the reanalysis of raw sequencing data available from public repositories. In particular, the new sequences we identified could be used in more in-depth analyses aiming to detect plasmids by recruiting unassembled sequencing reads from genome databases.

In *w*AlbA, pWALBA1 and pWALBA2 were maintained as multiple copies per *Wolbachia* cell, however, pWALBA1 copy number was relatively stable while pWALBA2 copy number increased with the age of female mosquitoes. Sequencing depth analysis indicated that other *Wolbachia*-associated plasmids also tend to be maintained as multi-copy plasmids, similar to what was observed in [23]. We found large variation in size among the complete circular plasmid genomes, ranging from ~9,000 to 21,000 bp, which is primarily driven by differences in the presence/absence and pseudogenization of accessory genes. Nevertheless, all plasmids appear to share a set of core genes likely to be essential to their maintenance. The *ParA*-like and downstream *ParG*-like genes have strong structural similarities with proteins involved in plasmid partition systems that ensure plasmid inheritance during cell division [51]. *ParA* family proteins are ATPases that drive the segregation of newly replicated plasmids into daughter cells [52] while *ParG* is known to act as a centromere-binding protein modulating the action of its cognate ATPase [53]. All plasmids also carry one or several *RelB/E* addiction modules which are Type II toxin-antitoxin (TA) systems that can promote plasmid maintenance through post-segregational killing of plasmid-free daughter cells [54]. *RelB/E* modules are also widespread in bacterial chromosomes, including in *Wolbachia* within and outside of prophage regions [47,55]. The role of chromosomal TA systems is still being debated but they have been implicated in various functions such as stress-response [56], anti-phage defences [57] and may also prevent the loss of large genomic regions under fluctuating selection regimes [58]. *RelB/E* modules may also accumulate as selfish DNA in bacterial chromosomes without providing fitness benefits due to their addictive nature [55].

A striking feature of some of the *Wolbachia* plasmids is the presence of several phage-like genes raising the possibility that they produce virus particles and could be transmitted horizontally via transduction. The two DNA packaging genes encoding the terminase large subunit and portal protein may allow plasmid genomes to be incorporated into viral capsids, while the presence of a phage stabilization and a putative coat protein indicate that plasmids might encode their own capsid structural proteins. We did not detect phage tail genes; however, such genes could be among the hypothetical proteins present on the plasmids or tail-less virus particles could be produced instead. Another hypothesis is that plasmids may highjack the tail proteins of WO phage, although this is perhaps unlikely since plasmid-encoded DNA packaging and structural phage-like proteins are not closely-related to WO homologues. It is however interesting to note that several plasmid genes have homologues in WO prophage regions (addiction modules, *cif* genes, non-*cif* PD-(D/E)XK nuclease) and that in two *Wolbachia* genomes, plasmid-like islands are located next to phage regions suggesting that plasmids may sometimes replicate and/or be packaged along with WO phage genomes. The existence of extrachromosomal phages, maintained as circular plasmids has been known for decades [59–61] and a recent computational analysis revealed that such phage-plasmids are widespread in bacteria and encompass genetically diverse groups of mobile genetic elements [62]. Whether *Wolbachia*-associated plasmids produce phage particles remains to be investigated.

While being genetically related, not all *Wolbachia* plasmids carry phage-like genes. This could mean that they rely solely on vertical transmission as we did not find that they encode their own conjugative machinery. There is evidence that some plasmids and phage-inducible chromosomal islands can undergo transduction by hijacking and even remodelling the capsid of coinfecting bacteriophages [63–65]. Thus, it is possible that plasmids not carrying phage genes behave like phage satellites by being packaged into virions produced by WO prophage regions or by another coinfecting plasmid such as pWALBA1.

Over long evolutionary times, some *Wolbachia*-associated plasmids become integrated into the bacterial chromosome, a process that could be facilitated by the presence of many transposase sequences in plasmids and *Wolbachia* genomes. It also appears that both plasmids and chromosomal islands rapidly go extinct as indicated by their patchy distribution among *Wolbachia* strains and the abundance of pseudogenes. Their long-term persistence may therefore depend on a positive balance between new acquisitions through horizontal transfers of plasmids and losses through intense pseudogenization prior to or following an integration into the bacterial chromosome. The presence of RelB/E addiction modules in plasmids and islands likely contributes to lower the speed at which they become lost by preventing large deletions and plasmid losses during cell division. Selection may also act to maintain some of the plasmid/island genes if they confer benefits to *Wolbachia*. In particular, it is of interest that some *cif* genes are found on plasmids in both *Wolbachia* and *Rickettsia*. Although the function of plasmid-encoded *cif* proteins remains to be tested experimentally, they may provide the ability to induce CI or other reproductive phenotypes such as parthenogenesis [34] and therefore contribute to the spread of the symbiont through host populations. There may even be an advantage for the symbiont in carrying *cif* genes on multi-copy plasmids rather than in the chromosome if an increase in the expression level of the cif proteins can modulate the CI phenotype and its rescue. Importantly, we showed that beyond phage WO, the *cif* genes can be associated with other mobile genetic elements like plasmids and integrative conjugal elements which likely contributed to their early evolution and spread across alphaproteobacterial symbionts.

In conclusion, our discovery of new plasmids associated with *w*AlbA and other *Wolbachia* strains provides a new framework for studying the role of these mobile genetic elements in *Wolbachia*. Importantly, our comparative analysis will guide future attempts aiming to develop a genetic tool kit for *Wolbachia* transformation. In particular, we expect that successful plasmid constructs will likely require some of the core genes we characterized such as those of the plasmid partition system and addiction modules.

## Material and methods

### Generation of a *w*AlbA-infected mosquito line

The *Ae*. *albopictus* wild type strain used in this study was collected from the Jalan Fletcher area of Kuala Lumpur (JF), Malaysia, in 2017. To generate a *w*AlbA-single infection, wild-type (*w*AlbA/*w*AlbB-carrying) larvae were reared under conditions of heat stress (diurnal cycle: 12hr at 37°C / 12 hr at 32°C). Twenty-two resulting adult females were mated en-masse to males of a previously tetracycline-treated *Wolbachia*-negative JF line. Eleven of these successfully blood-fed and were individualised for oviposition. Viable egg batches were obtained from 7 females, which were subsequently hatched independently. Resulting male pupae were screened for *w*AlbA and *w*AlbB by strain specific PCR primers (S5 Table). Two of the 7 sibling pools contained males with a positive signal for *w*AlbA and no signal for *w*AlbB, with the remaining 5 pools giving no signal for either strain. Female siblings from the wAlbA-positive pools were crossed to males of the *Wolbachia*-negative JF line, blood-fed and individualised for oviposition. An isofemale line carrying only *w*AlbA was isolated. Females from this line were subsequently outcrossed to *Wolbachia*-negative males from the JF line for five consecutive generations before a stable self-crossed colony was established.

*Wolbachia* density was measured in the dissected tissues of 5-day old adult females by quantitative PCR (qPCR) using *w*AlbA-specific primers (S5 Table) normalised to the host homothorax (HTH) gene using a SYBR Green qPCR master mix (Bimake), with the following conditions: 95°C for 5 mins, followed by 40 cycles of 95°C for 15 secs and 60°C for 60 secs, and subsequent melt curve analysis.

Fecundity of females of the wild-type, *w*AlbA-only and *Wolbachia*-negative lines was measured by crossing females of each line to *Wolbachia-*negative males in pools of 10 males to 5 females, followed by blood-feeding and selection of engorged females and subsequent individualisation of gravid females for oviposition on a damp filter-paper substrate. Egg batch per female was counted manually using a dissection microscope.

Levels of cytoplasmic incompatibility induction and rescue were measured by crossing various combinations of males and females from the wild-type, *w*AlbA-only and *Wolbachia*-negative lines in pools of 10 males to 5 females, followed by flood-feeding and individualisation as described above. Female mating status post-oviposition was assessed by dissection of spermathecae and visual confirmation of sperm transfer using an inverted 20x compound microscope. The eggs of females lacking visible sperm in spermathecae were discarded from further analysis. Eggs on filter papers from individual females were counted and dried for 5-days, prior to subsequent hatching in water containing bovine liver powder. L1 larval numbers were counted on emergence.

### *Wolbachia* purification and whole-genome amplification

For Illumina sequencing, *w*AlbA was purified by dissecting and pooling ovaries from 27 *w*AlbA-infected *Ae*. *albopictus* females. Pooled ovaries were rinsed in PBS three times, resuspended in Schneider’s media and homogenized with a sterile pestle. The homogenate was centrifuged at 2,000 g for 2 min to remove cellular debris and the supernatant was filtered through 5 and 2.7 μm sterile filters. The filtrate was centrifuged at 18,500 g for 15 min. The bacterial pellet was resuspended in Schneider’s media and treated with DNase I at 37°C for 30 min to remove host DNA. Following digestion, the DNase was inactivated at 75°C for 10 min and the sample centrifuged at 18,500 g to discard the supernatant. DNA was amplified directly from the bacterial pellet using the REPLI-G Midi kit (Qiagen). The whole-genome amplified DNA (WGA) was cleaned using the QIAamp DNA mini kit (Qiagen). For Oxford Nanopore Technology (ONT) sequencing, ovaries from 100 females were dissected and pooled for DNA extraction as above except that no *Wolbachia* purification or whole-genome amplification was conducted (non-WGA). Finally, DNA was extracted from another pool of 23 pairs of ovaries without WGA to conduct an additional run of Illumina sequencing. However, this sample did not allow us to complete the *w*AlbA genome. Therefore, the corresponding Illumina data was only used for analysing variation in sequencing depth (see below) since reads mapped onto the *w*AlbA genome and plasmids were more evenly distributed than for the WGA sample due to amplification bias.

### Genome sequencing and de novo assembly

A DNA library was prepared from the WGA sample using the Kapa LTP Library Preparation Kit (KAPA Biosystems, Roche7961880001) and sequenced on the Illumina MiSeq platform with the MiSeq Reagent Kit v3 to generate 2×150 bp paired-end reads. The non-WGA sample was used to prepare an ONT library by shearing the DNA into ~8 kb fragments followed by purification and size-selection using AMPure XP beads (Beckman Coulter). The ONT library was then prepared with the Ligation Sequencing Kit (SQK-LSK109) and sequenced for 72 hours using a GridION (ONT) controlled by the MinKNOW software v20.06.9. Illumina and ONT adapters were removed with Trimmomatic v0.38.0 [66] and Porechop v0.2.4 [67] respectively. Mosquito reads were filtered out by mapping the Illumina and ONT reads against the *Ae*. *albopictus* reference assembly (Genbank accession: GCF_006496715.1) using Bowtie2 v2.4.2 [68] and Minimap2 v2.23 [69] respectively. Unmapped reads were then assembled using the Unicycler hybrid assembly pipeline which assesses the circularity of assembled replicons based on the presence/absence of an end in the assembly graph structure [70]. Circular genome maps were created in DNAPlotter [71].

### Endpoint PCR and quantitative PCR

Genomic DNA was extracted by homogenizing individual female mosquitoes in 200 μL STE buffer with a sterile pestle, followed by a 30 min incubation at 65°C with 2 μL of Proteinase K (20 mg/mL) and a final incubation for 10 min at 95°C. The extracted DNA was diluted 1/5 in water and samples were centrifuged at 2,000 g for 2 min before PCR. For each PCR reaction, 2 μL of DNA template was amplified using the 2x Taq master mix (Vazyme) in a 25 μL reaction: 12.5 μL of master mix, 12.5 μL of water, 1 μL of each 10 μM primer (S5 Table) and the following PCR cycles: 95°C for 3 min, 35 cycles of 15s denaturation at 95°C, 15s for primer annealing (see temperatures in S5 Table), 1 min extension at 72°C and a 5 min final extension step at 72°C. *Wolbachia* density relative to host DNA and plasmid copy number were measured by qPCR using the QuantiNova SYBR Green PCR kit (Qiagen) in 10 μL reactions: 5 μL of master mix, 2 μL of water, 0.5 μL of each 5 μM primer (S5 Table) and the following cycles: 95°C for 15 min, 40× cycles of 95°C for 15 s and 60°C for 20 s, followed by a melt-curve analysis.

### Search of plasmid sequences in publicly available sequencing data

The presence of plasmid sequences in publicly available *Wolbachia* genomes was searched with TBLASTN [72] using amino acid sequences of all pWALBA1 and pWALBA2 genes. Default parameters and an e-value threshold of 0.05 were used. TBLASTN hits were then visually inspected in Artemis [73]. When raw sequencing data was available from the Sequence Read Archive (https://www.ncbi.nlm.nih.gov/sra), we attempted to circularize plasmid-like sequences by conducting de novo assemblies using Unicycler. In the case of the *w*Con plasmid, circularization was achieved by visualizing the final assembly graph in Bandage [74] and removing an ambiguous path that linked the plasmid to a short multicopy transposase sequence also present in the *Wolbachia* genome (40x sequencing depth relative to the bacterial chromosome). Circular plasmid sequences were then manually rotated to start from the *ParA*-like plasmid partition gene. pWALBA1 and pWALBA2 amino acid sequences were also individually used as queries in the BLASTP online tool (https://blast.ncbi.nlm.nih.gov/Blast.cgi; last accessed January, 2022) to find additional plasmid-like sequences in genome assemblies not annotated as *Wolbachia*. Homologues of the *cif* genes in *Wolbachia* and other *Rickettsiales* genomes were also detected using TBLASTN and representative sequences of the five *cif* types as explained in [33].

In order to investigate gene function and synteny conservation between plasmid sequences, all sequences were reannotated using the RAST annotation pipeline [75] and putative pseudogenes were manually corrected in Artemis. The function of the predicted coding sequences was also inferred from BLASTP searches and using the HHpred webserver [76] against the following databases: SCOPe70 (v2.07), Pfam (v35), SMART (v6.0), and COG/KOG (v1.0). Annotated plasmid sequences were then plotted side-by-side along with BLASTN similarities using the R package GenoPlotR [77].

Finally, plasmid copy number relative to the *Wolbachia* genome was estimated by mapping the raw sequencing reads onto their respective genome assembly using Bowtie2 v2.4.2 [68] and extracting the sequencing depth values at each position with the Samtools depth command [78]. Sequencing depth per 500 bp windows was calculated and visualized in the R software with a custom script [79]. A more detailed analysis of sequencing depth and clonality of the *w*AlbA plasmids was also carried out using anvi’o v7.1 [80].

### Phylogenetic analysis

The *Wolbachia* phylogeny was generated with RAxML v7.7.6 [81] based on the concatenated nucleotide alignment of 36 core genes selected from a set of orthologous single-copy genes described in [82]. For the plasmid and *cif* genes, nucleotide sequences were aligned with MAFFT using the codon-aware tool of the TranslatorX webserver [83] after manually removing premature stop codons from pseudogene sequences. Poorly aligned regions were then trimmed from the alignments using trimAl v1.3 with the–automated1 method [84]. PhyML v3.0 [85] was used to build gene phylogenies with the GTR GAMMA substitution model and 100 bootstrap replicates. Trees were annotated in the iTOL online tool [86] and the cophylo function from the phytools R package [87] for the plasmid and *cif* genes respectively.

## Supporting information

S1 Fig *w*AlbA tissue tropism and fitness effects. (A) *w*AlbA relative density within tissues of individual females. (B) Number of eggs laid by individual females. (C) Egg hatch rates in the progeny of individual females. Blue, red and grey indicate doubly-infected, *w*AlbA-infected and *Wolbachia*-free mosquito lines respectively. NS: non-significant; *: p < 0.05; **: p < 0.01; ***: p < 0.001.
(JPEG)

S2 Fig Sequencing depth and clonality analysis of *w*AlbA plasmid genomes. (TIF)

S3 Fig Endpoint PCR targeting *w*AlbA and its associated plasmids. DNA was extracted from individual female mosquitoes and from the Aa23 cell line (n = 4 per *Wolbachia* infection status). Nomenclature: host background-*Wolbachia* infection status. NC: PCR negative control. L: DNA ladder.
(JPEG)

S4 Fig Quantitative PCR analysis of *w*AlbA plasmid copy numbers. DNA was extracted from individual *w*AlbA-infected female mosquitoes at different timepoints. Females were blood-fed at 8 and 18 day-old. (A) Copy number of a plasmid-specific target (*DnaB*-like gene and Fic family protein for pWALBA1 and pWALBA2 respectively) relative to *Wolbachia* 16S rRNA copies. The *p*-values were calculated with a *t* test on paired log-transformed data. (B) Correlation between *w*AlbA densities and plasmid copy number per mosquito (blue: pWALBA1, red: pWALBA2). The dashed lines show predicted values from linear regressions and *r* is the Pearson’s correlation coefficient.
(JPEG)

S5 Fig Putative chimeric spider contigs. *A*. *araneus* (A) and *T*. *clavipes* (B) contigs were blasted against their respective plasmid-like region, the *w*Mel (Supergroup A) and *w*AlbB (Supergroup B) reference genomes. BLASTN hits were visualized in Bandage. Sequencing depth was measured by mapping the Illumina and Nanopore reads from the corresponding sample onto the contig of interest.
(JPEG)

S6 Fig Phylogenies of *ParA*-like, *ParG*-like, HP1 and *DnaB*-like homologues. Full circles indicate the genomic location of the different homologues. Branch support calculated from 100 bootstraps replicates and >80% are shown.
(JPEG)

S7 Fig Phylogenies of phage portal, terminase large subunit, stabilization and coat protein homologues. Full circles indicate the genomic location of the different homologues. Branch support calculated from 100 bootstraps replicates and >80% are shown.
(JPEG)

S8 Fig Phylogenies of HP2, HP3, HP4 and *QueT*-like transporter protein homologues. Full circles indicate the genomic location of the different homologues. Branch support calculated from 100 bootstraps replicates and >80% are shown.
(JPEG)

S9 Fig Phylogenies of HP7, HP9, PD-(D/E)XK nuclease and Fic/DOC protein homologues. Full circles indicate the genomic location of the different homologues. Branch support calculated from 100 bootstraps replicates and >80% are shown.
(JPEG)

S10 Fig Phylogenies of *RelB* and *RelE* homologues. Full circles indicate the genomic location of the different homologues. Branch support calculated from 100 bootstraps replicates and >80% are shown.
(JPEG)

S11 Fig Association of *cif* genes with Integrative Conjugative elements. Similarity is indicated by gene colours (BLASTP) and by the grey areas between sequences (TBLASTX) where darker grey means more similar. Dark grey genes are transposable element sequences.
(JPEG)

S1 Table *Wolbachia* genome statistics. (XLSX)

S2 Table *w*AlbA prophage regions. (XLSX)

S3 Table Plasmid gene BLASTP hits and HHpred predicted domains. (XLSX)

S4 Table List of *Wolbachia* genome assemblies and detected plasmid-like regions. (XLSX)

S5 Table List of PCR and qPCR primers. (XLSX)

## References

[1] Weinert LA, Araujo-Jnr E V., Ahmed MZ, Welch JJ. The incidence of bacterial endosymbionts in terrestrial arthropods. Proc R Soc B Biol Sci. 2015;282: 20150249–20150249. doi: 10.1098/rspb.2015.0249 25904667 PMC4424649

[2] Taylor MJ, Bandi C, Hoerauf A. Wolbachia bacterial endosymbionts of filarial nematodes. Adv Parasitol. 2005;60: 245–284. doi: 10.1016/S0065-308X(05)60004-8 16230105

[3] Kaur R, Shropshire JD, Cross KL, Leigh B, Mansueto AJ, Stewart V, et al. Living in the endosymbiotic world of Wolbachia: A centennial review. Cell Host Microbe. 2021;29: 879–893. doi: 10.1016/j.chom.2021.03.006 33945798 PMC8192442

[4] Martinez J, Longdon B, Bauer S, Chan Y-S, Miller WJ, Bourtzis K, et al. Symbionts Commonly Provide Broad Spectrum Resistance to Viruses in Insects: A Comparative Analysis of Wolbachia Strains. PLoS Pathog. 2014;10: e1004369. doi: 10.1371/journal.ppat.1004369 25233341 PMC4169468

[5] Teixeira L, Ferreira A, Ashburner M. The Bacterial Symbiont Wolbachia Induces Resistance to RNA Viral Infections in Drosophila melanogaster. Plos Biol. 2008;6: 2753–2763. doi: 10.1371/journal.pbio.1000002 19222304 PMC2605931

[6] Ant TH, Herd CS, Geoghegan V, Hoffmann AA, Sinkins SP. The Wolbachia strain wAu provides highly efficient virus transmission blocking in Aedes aegypti. PLOS Pathog. 2018;14: e1006815. doi: 10.1371/journal.ppat.1006815 29370307 PMC5784998

[7] Walker T, Johnson PH, Moreira LA, Iturbe-Ormaetxe I, Frentiu FD, McMeniman CJ, et al. The wMel Wolbachia strain blocks dengue and invades caged Aedes aegypti populations. Nature. 2011;476: 450–3. doi: 10.1038/nature10355 21866159

[8] Nazni WA, Hoffmann AA, NoorAfizah A, Cheong YL, Mancini M V, Golding N, et al. Establishment of Wolbachia Strain wAlbB in Malaysian Populations of Aedes aegypti for Dengue Control. Curr Biol. 2019/11/21. 2019;29: 4241–4248.e5. doi: 10.1016/j.cub.2019.11.007 31761702 PMC6926472

[9] Utarini A, Indriani C, Ahmad RA, Tantowijoyo W, Arguni E, Ansari MR, et al. Efficacy of Wolbachia-Infected Mosquito Deployments for the Control of Dengue. N Engl J Med. 2021;384: 2177–2186. doi: 10.1056/NEJMoa2030243 34107180 PMC8103655

[10] Gesto JSM, Ribeiro GS, Rocha MN, Dias FBS, Peixoto J, Carvalho FD, et al. Reduced competence to arboviruses following the sustainable invasion of Wolbachia into native Aedes aegypti from Southeastern Brazil. Sci Rep. 2021;11: 10039. doi: 10.1038/s41598-021-89409-8 33976301 PMC8113270

[11] Wu M, Sun L V, Vamathevan J, Riegler M, Deboy R, Brownlie JC, et al. Phylogenomics of the reproductive parasite Wolbachia pipientis wMel: a streamlined genome overrun by mobile genetic elements. PLoS Biol. 2004;2: E69. doi: 10.1371/journal.pbio.0020069 15024419 PMC368164

[12] Chrostek E, Marialva MSP, Esteves SS, Weinert LA, Martinez J, Jiggins FM, et al. Wolbachia Variants Induce Differential Protection to Viruses in Drosophila melanogaster: A Phenotypic and Phylogenomic Analysis. PLoS Genet. 2013;9: e1003896. doi: 10.1371/journal.pgen.1003896 24348259 PMC3861217

[13] Gerth M, Bleidorn C, Weinert LA, Araujo-Jnr E V., Ahmed MZ, Welch JJ, et al. Comparative genomics provides a timeframe for Wolbachia evolution and exposes a recent biotin synthesis operon transfer. Nat Microbiol. 2017;2: 16241. doi: 10.1038/nmicrobiol.2016.241 28005061

[14] Ellegaard KM, Klasson L, Näslund K, Bourtzis K, Andersson SGE. Comparative genomics of Wolbachia and the bacterial species concept. PLoS Genet. 2013;9: e1003381. doi: 10.1371/journal.pgen.1003381 23593012 PMC3616963

[15] Scholz M, Albanese D, Rota-stabelli O, Donati C, Segata N. Large scale genome reconstructions illuminate Wolbachia evolution. Nat Commun. 2020;11: 535. doi: 10.1038/s41467-020-19016-0 33067437 PMC7568565

[16] Bordenstein SR, Bordenstein SR. Widespread phages of endosymbionts: Phage WO genomics and the proposed taxonomic classification of Symbioviridae. PLoS Genet. 2022;18: e1010227. doi: 10.1371/journal.pgen.1010227 35666732 PMC9203015

[17] Lepage DP, Metcalf JA, Bordenstein SR, On J, Perlmutter JI, Shropshire JD, et al. Prophage WO genes recapitulate and enhance Wolbachia-induced cytoplasmic incompatibility. Nature. 2017;9: 243–247. doi: 10.1038/nature21391 28241146 PMC5358093

[18] Beckmann J, Ronau J, Hochstrasser M. A wolbachia deubiquitylating enzyme induces cytoplasmic incompatibility. Nat Microbiol. 2017;2: 17007. doi: 10.1038/nmicrobiol.2017.7 28248294 PMC5336136

[19] Bordenstein SR, Bordenstein SR. Eukaryotic association module in phage WO genomes from Wolbachia. Nat Commun. 2016;7: 1–10. doi: 10.1038/ncomms13155 27727237 PMC5062602

[20] Kupritz J, Martin J, Fischer K, Curtis KC, Fauver JR, Huang Y, et al. Isolation and characterization of a novel bacteriophage WO from Allonemobius socius crickets in Missouri. PLoS One. 2021;16: 1–17. doi: 10.1371/journal.pone.0250051 34197460 PMC8248633

[21] Masui S, Kuroiwa H, Sasaki T, Inui M, Kuroiwa T, Ishikawa H. Bacteriophage WO and Virus-like Particles in Wolbachia, an Endosymbiont of Arthropods. Biochem Biophys Res Commun. 2001;283: 1099–1104. doi: 10.1006/bbrc.2001.4906 11355885

[22] Fujii Y, Kubo T, Ishikawa H, Sasaki T. Isolation and characterization of the bacteriophage WO from Wolbachia, an arthropod endosymbiont. Biochem Biophys Res Commun. 2004;317: 1183–1188. doi: 10.1016/j.bbrc.2004.03.164 15094394

[23] Reveillaud J, Bordenstein SR, Cruaud C, Shaiber A, Esen ÖC, Weill M, et al. The Wolbachia mobilome in Culex pipiens includes a putative plasmid. Nat Commun. 2019;10: 1051. doi: 10.1038/s41467-019-08973-w 30837458 PMC6401122

[24] Norman A, Hansen LH, Sørensen SJ. Conjugative plasmids: Vessels of the communal gene pool. Philos Trans R Soc B Biol Sci. 2009;364: 2275–2289. doi: 10.1098/rstb.2009.0037 19571247 PMC2873005

[25] Bennett PM. Plasmid encoded antibiotic resistance: acquisition and transfer of antibiotic resistance genes in bacteria. Br J Pharmacol. 2008;153: S347–S357. doi: 10.1038/sj.bjp.0707607 18193080 PMC2268074

[26] Rodríguez-Rubio L, Serna C, Ares-Arroyo M, Matamoros BR, Delgado-Blas JF, Montero N, et al. Extensive antimicrobial resistance mobilization via multicopy plasmid encapsidation mediated by temperate phages. J Antimicrob Chemother. 2020;75: 3173–3180. doi: 10.1093/jac/dkaa311 32719862 PMC7566468

[27] Santos-Garcia D, Rollat-Farnier PA, Beitia F, Zchori-Fein E, Vavre F, Mouton L, et al. The genome of Cardinium cBtQ1 provides insights into genome reduction, symbiont motility and its settlement in Bemisia tabaci. Genome Biol Evol. 2014;6: 1013–1030. doi: 10.1093/gbe/evu077 24723729 PMC4007549

[28] Penz T, Schmitz-Esser S, Kelly SE, Cass BN, Müller A, Woyke T, et al. Comparative Genomics Suggests an Independent Origin of Cytoplasmic Incompatibility in Cardinium hertigii. Moran NA, editor. PLoS Genet. 2012;8: e1003012. doi: 10.1371/journal.pgen.1003012 23133394 PMC3486910

[29] Masson F, Copete SC, Schüpfer F, Garcia-Arraez G, Lemaitre B. In Vitro culture of the insect Endosymbiont Spiroplasma poulsonii highlights bacterial genes involved in host- symbiont interaction. MBio. 2018;9: 1–11. doi: 10.1128/mBio.00024-18 29559567 PMC5874924

[30] Pollmann M, Moore LD, Krimmer E, Matthew J. Highly transmissible cytoplasmic incompatibility by the extracellular insect symbiont Spiroplasma by the extracellular insect symbiont Spiroplasma. ISCIENCE. 2022;25: 104335. doi: 10.1016/j.isci.2022.104335 35602967 PMC9118660

[31] Gillespie JJ, Driscoll TP, Verhoeve VI, Utsuki T, Husseneder C, Chouljenko VN, et al. Genomic Diversification in Strains of Rickettsia felis Isolated from Different Arthropods. Genome Biol Evol. 2014;7: 35–56. doi: 10.1093/gbe/evu262 25477419 PMC4316617

[32] Harumoto T, Lemaitre B. Male-killing toxin in a bacterial symbiont of Drosophila. Nature. 2018;557: 252–255. doi: 10.1038/s41586-018-0086-2 29720654 PMC5969570

[33] Martinez J, Klasson L, Welch JJ, Jiggins FM. Life and death of selfish genes: comparative genomics reveals the dynamic evolution of cytoplasmic incompatibility. Mol Biol Evol. 2021;38: 2–15. doi: 10.1093/molbev/msaa209 32797213 PMC7783169

[34] Gillespie JJ, Driscoll TP, Verhoeve VI, Rahman MS, Kevin R, Azad AF. A Tangled Web: Origins of Reproductive Parasitism. Genome Biol Evol. 2018;10: 2292–2309. doi: 10.1093/gbe/evy159 30060072 PMC6133264

[35] Dutton TJ, Sinkins SP. Strain-specific quantification of Wolbachia density in Aedes albopictus and effects of larval rearing conditions. Insect Mol Biol. 2004;13: 317–322. doi: 10.1111/j.0962-1075.2004.00490.x 15157232

[36] Ant TH, Sinkins SP. A Wolbachia triple-strain infection generates self-incompatibility in Aedes albopictus and transmission instability in Aedes aegypti. Parasites and Vectors. 2018;11: 1–7. doi: 10.1186/s13071-018-2870-0 29751814 PMC5948879

[37] Driscoll TP, Verhoeve VI, Brockway C, Shrewsberry DL, Plumer M, Sevdalis SE, et al. Evolution of Wolbachia mutualism and reproductive parasitism: insight from two novel strains that co-infect cat fleas. PeerJ. 2020;8: e10646. doi: 10.7717/peerj.10646 33362982 PMC7750005

[38] Jones MW, Fricke LC, Thorpe CJ, Vander Esch LO, Lindsey ARI. Infection Dynamics of Cotransmitted Reproductive Symbionts Are Mediated by Sex, Tissue, and Development. Appl Environ Microbiol. 2022;88: 1–12. doi: 10.1128/aem.00529-22 35730939 PMC9275221

[39] Sinha A, Li Z, Sun L, Carlow CKS, Cordaux R. Complete Genome Sequence of the Wolbachia wAlbB Endosymbiont of Aedes albopictus. Genome Biol Evol. 2019;11: 706–720. doi: 10.1093/gbe/evz025 30715337 PMC6414309

[40] Ross PA, Gu X, Robinson KL, Yang Q, Cottingham E, Zhang Y, et al. A wAlbB Wolbachia Transinfection Displays Stable Phenotypic Effects across Divergent Aedes aegypti Mosquito Backgrounds. Appl Environ Microbiol. 2021;87: e01264–21. doi: 10.1128/AEM.01264-21 34379518 PMC8478461

[41] Pascar J, Chandler CH. A bioinformatics approach to identifying Wolbachia infections in arthropods. PeerJ. 2018;6: e5486. doi: 10.7717/peerj.5486 30202647 PMC6126470

[42] Surendra N, I. BS, M. MA, S. P-SK, G. NIL. Near-Complete Genome Sequences of a Wolbachia Strain Isolated from Diaphorina citri Kuwayama (Hemiptera: Liviidae). Microbiol Resour Announc. 2022;9: e00560–20. doi: 10.1128/MRA.00560-20 32855244 PMC7453280

[43] Baião GC, Janice J, Galinou M, Klasson L. Comparative genomics reveals factors associated with phenotypic expression of Wolbachia. Genome Biol Evol. 2021;13: 1–20. doi: 10.1093/gbe/evab111 34003269 PMC8290115

[44] Steczkiewicz K, Muszewska A, Knizewski L, Rychlewski L, Ginalski K. Sequence, structure and functional diversity of PD-(D/E)XK phosphodiesterase superfamily. Nucleic Acids Res. 2012;40: 7016–7045. doi: 10.1093/nar/gks382 22638584 PMC3424549

[45] Fallon AM. Muramidase, nuclease, or hypothetical protein genes intervene between paired genes encoding DNA packaging terminase and portal proteins in Wolbachia phages and prophages. Virus Genes. 2022;58: 327–349. doi: 10.1007/s11262-022-01907-7 35538383

[46] Halter T, Köstlbacher S, Rattei T, Hendrickx F, Manzano-marín A, Horn M. One to host them all: genomics of the diverse bacterial endosymbionts of the spider Oedothorax gibbosus. bioRxiv. 2022; 494226. 10.1099/mgen.0.000943 PMC9997750 36757767

[47] Fallon AM. Computational evidence for antitoxins associated with RelE/ParE, RatA, Fic, and AbiEii-family toxins in Wolbachia genomes. Mol Genet Genomics. 2020;295: 891–909. doi: 10.1007/s00438-020-01662-0 32189066

[48] Takano S, Gotoh Y, Hayashi T. “Candidatus Mesenet longicola”: Novel Endosymbionts of Brontispa longissima that Induce Cytoplasmic Incompatibility. Microb Ecol. 2021;82: 512–522. doi: 10.1007/s00248-021-01686-y 33454808

[49] Davison HR, Pilgrim J, Wybouw N, Parker J, Pirro S, Hunter-Barnett S, et al. Large-scale comparative genomics unravels great genomic diversity across the Rickettsia and Ca. Megaira genera and identifies Torix group as an evolutionarily distinct clade. bioRxiv. 2021; 2021.10.06.463315.

[50] Akayama KN, Amashita AY, Urokawa KK, Orimoto TM, Gawa MO, Ukuhara MF, et al. The Whole-genome Sequencing of the Obligate Intracellular Bacterium Orientia tsutsugamushi Revealed Massive Gene Amplification During Reductive Genome Evolution. DNA Res. 2008;15: 185–199. doi: 10.1093/dnares/dsn011 18508905 PMC2575882

[51] Baxter JC, Funnell BE. Plasmid Partition Mechanisms. Microbiol Spectr. 2014;2: PLAS-0023-2014. doi: 10.1128/microbiolspec.PLAS-0023-2014 26104442

[52] Radnedge L, Youngren B, Davis M, Austin S. Probing the structure of complex macromolecular interactions by homolog specificity scanning: the P1 and P7 plasmid partition systems. EMBO J. 1998;17: 6076–6085. doi: 10.1093/emboj/17.20.6076 9774351 PMC1170934

[53] Wu M, Zampini M, Bussiek M, Hoischen C, Diekmann S, Hayes F. Segrosome assembly at the pliable parH centromere. Nucleic Acids Res. 2011;39: 5082–5097. doi: 10.1093/nar/gkr115 21378121 PMC3130281

[54] Gotfredsen M, Gerdes K. The Escherichia coli relBE genes belong to a new toxin–antitoxin gene family. Mol Microbiol. 1998;29: 1065–1076. doi: 10.1046/j.1365-2958.1998.00993.x 9767574

[55] Fraikin N, Goormaghtigh F, van Melderen L. Type II toxin-antitoxin systems: Evolution and revolutions. J Bacteriol. 2020;202. doi: 10.1128/JB.00763-19 31932311 PMC7167474

[56] Christensen S, Mikkelsen M, Pedersen K, Gerdes K. RelE, a global inhibitor of translation, is activated during nutritional stress. Proc Natl Acad Sci. 2001;98: 14328–14333. doi: 10.1073/pnas.251327898 11717402 PMC64681

[57] Hazan R, Engelberg-Kulka H. Escherichia coli mazEF-mediated cell death as a defense mechanism that inhibits the spread of phage P1. Mol Genet Genomics. 2004;272: 227–234. doi: 10.1007/s00438-004-1048-y 15316771

[58] Szekeres S, Dauti M, Wilde C, Mazel D, Rowe-Magnus DA. Chromosomal toxin–antitoxin loci can diminish large-scale genome reductions in the absence of selection. Mol Microbiol. 2007;63: 1588–1605. doi: 10.1111/j.1365-2958.2007.05613.x 17367382

[59] Łobocka MB, Rose DJ, Plunkett G 3rd, Rusin M, Samojedny A, Lehnherr H, et al. Genome of bacteriophage P1. J Bacteriol. 2004;186: 7032–7068. doi: 10.1128/JB.186.21.7032-7068.2004 15489417 PMC523184

[60] Utter B, Deutsch DR, Schuch R, Winer BY, Verratti K, Bishop-Lilly K, et al. Beyond the Chromosome: The Prevalence of Unique Extra-Chromosomal Bacteriophages with Integrated Virulence Genes in Pathogenic Staphylococcus aureus. PLoS One. 2014;9: e100502. doi: 10.1371/journal.pone.0100502 24963913 PMC4070920

[61] Gilcrease EB, Casjens SR. The genome sequence of Escherichia coli tailed phage D6 and the diversity of Enterobacteriales circular plasmid prophages. Virology. 2018;515: 203–214. doi: 10.1016/j.virol.2017.12.019 29304472 PMC5800970

[62] Pfeifer E, Moura De Sousa JA, Touchon M, Rocha EPC. Bacteria have numerous distinctive groups of phage-plasmids with conserved phage and variable plasmid gene repertoires. Nucleic Acids Res. 2021;49: 2655–2673. doi: 10.1093/nar/gkab064 33590101 PMC7969092

[63] Fillol-salom A, Bacarizo J, Alqasmi M, Chen J, Penade R, Marina A. Hijacking the Hijackers: Escherichia coli Pathogenicity Islands Redirect Helper Phage Packaging for Their Own Benefit Article Hijacking the Hijackers: Escherichia coli Pathogenicity Islands Redirect Helper Phage Packaging for Their Own Benefit. Mol Cell. 2019;75: 1020–1030. doi: 10.1016/j.molcel.2019.06.017 31350119 PMC6739421

[64] Humphrey S, San Millán Á, Toll-Riera M, Connolly J, Flor-Duro A, Chen J, et al. Staphylococcal phages and pathogenicity islands drive plasmid evolution. Nat Commun. 2021;12: 1–15. doi: 10.1038/s41467-021-26101-5 34615859 PMC8494744

[65] Penadés JR, Christie GE. The Phage-Inducible Chromosomal Islands: A Family of Highly Evolved Molecular Parasites. Annu Rev Virol. 2015;2: 181–201. doi: 10.1146/annurev-virology-031413-085446 26958912

[66] Bolger AM, Lohse M, Usadel B. Trimmomatic: A flexible trimmer for Illumina sequence data. Bioinformatics. 2014;30: 2114–2120. doi: 10.1093/bioinformatics/btu170 24695404 PMC4103590

[67] Wick R. Porechop. GitHub repository. GitHub; 2017. Available: https://github.com/rrwick/Porechop

[68] Langmead B, Salzberg SL. Fast gapped-read alignment with Bowtie 2. Nat Methods. 2012;9: 357–9. doi: 10.1038/nmeth.1923 22388286 PMC3322381

[69] Li H. Minimap2: pairwise alignment for nucleotide sequences. Bioinformatics. 2018;34: 3094–3100. doi: 10.1093/bioinformatics/bty191 29750242 PMC6137996

[70] Wick RR, Judd LM, Gorrie CL, Holt KE. Unicycler: Resolving bacterial genome assemblies from short and long sequencing reads. PLOS Comput Biol. 2017;13: e1005595. doi: 10.1371/journal.pcbi.1005595 28594827 PMC5481147

[71] Carver T, Thomson N, Bleasby A, Berriman M, Parkhill J. DNAPlotter: circular and linear interactive genome visualization. Bioinformatics. 2009;25: 119–120. doi: 10.1093/bioinformatics/btn578 18990721 PMC2612626

[72] Altschul SF, Gish W, Miller W, Myers EW, Lipman DJ. Basic local alignment search tool. J Mol Biol. 1990;215: 403–410. doi: 10.1016/S0022-2836(05)80360-2 2231712

[73] Carver T, Harris SR, Berriman M, Parkhill J, McQuillan JA. Artemis: an integrated platform for visualization and analysis of high-throughput sequence-based experimental data. Bioinformatics. 2012;28: 464–469. doi: 10.1093/bioinformatics/btr703 22199388 PMC3278759

[74] Wick RR, Schultz MB, Zobel J, Holt KE. Bandage: interactive visualization of de novo genome assemblies. Bioinformatics. 2015;31: 3350–3352. doi: 10.1093/bioinformatics/btv383 26099265 PMC4595904

[75] Aziz RK, Bartels D, Best AA, DeJongh M, Disz T, Edwards RA, et al. The RAST Server: Rapid Annotations using Subsystems Technology. BMC Genomics. 2008;9: 75. doi: 10.1186/1471-2164-9-75 18261238 PMC2265698

[76] Söding J, Biegert A, Lupas AN. The HHpred interactive server for protein homology detection and structure prediction. Nucleic Acids Res. 2005/06/27. 2005;33: W244–W248. doi: 10.1093/nar/gki408 15980461 PMC1160169

[77] Guy L, Roat Kultima J, Andersson SGE. genoPlotR: comparative gene and genome visualization in R. Bioinformatics. 2010;26: 2334–2335. doi: 10.1093/bioinformatics/btq413 20624783 PMC2935412

[78] Li H, Handsaker B, Wysoker A, Fennell T, Ruan J, Homer N, et al. The Sequence alignment/map (SAM) format and SAMtools. Bioinformatics. 2009;25. doi: 10.1093/bioinformatics/btp352 19505943 PMC2723002

[79] R Core Team. R: A Language and Environment for Statistical Computing. Pimenta PF, editor. Vienna, Austria; 2013.

[80] Eren AM, Kiefl E, Shaiber A, Veseli I, Miller SE, Schechter MS, et al. Community-led, integrated, reproducible multi-omics with anvi’o. Nat Microbiol. 2021;6: 3–6. doi: 10.1038/s41564-020-00834-3 33349678 PMC8116326

[81] Stamatakis A. RAxML version 8: a tool for phylogenetic analysis and post-analysis of large phylogenies. Bioinformatics. 2014;30: 1312–1313. doi: 10.1093/bioinformatics/btu033 24451623 PMC3998144

[82] Comandatore F, Sassera D, Montagna M, Kumar S, Koutsovoulos G, Thomas G, et al. Phylogenomics and analysis of shared genes suggest a single transition to mutualism in Wolbachia of nematodes. Genome Biol Evol. 2013;5: 1668–1674. doi: 10.1093/gbe/evt125 23960254 PMC3787677

[83] Abascal F, Zardoya R, Telford MJ. TranslatorX: multiple alignment of nucleotide sequences guided by amino acid translations. Nucleic Acids Res. 2010;38: W7–13. doi: 10.1093/nar/gkq291 20435676 PMC2896173

[84] Capella-Gutiérrez S, Silla-Martínez JM, Gabaldón T. trimAl: a tool for automated alignment trimming in large-scale phylogenetic analyses. Bioinformatics. 2009;25: 1972–1973. doi: 10.1093/bioinformatics/btp348 19505945 PMC2712344

[85] Guindon S, Dufayard J-F, Lefort V, Anisimova M, Hordijk W, Gascuel O. New algorithms and methods to estimate maximum-likelihood phylogenies: assessing the performance of PhyML 3.0. Syst Biol. 2010;59: 307–321. doi: 10.1093/sysbio/syq010 20525638

[86] Letunic I, Bork P. Interactive Tree Of Life (iTOL): an online tool for phylogenetic tree display and annotation. Bioinformatics. 2007;23: 127–128. doi: 10.1093/bioinformatics/btl529 17050570

[87] Revell LJ. phytools: an R package for phylogenetic comparative biology (and other things). Methods Ecol Evol. 2012;3: 217–223. 10.1111/j.2041-210X.2011.00169.x

