# Supplementary figures and images for: Genome sequencing and comparative analysis of Wolbachia strain wAlbA reveals Wolbachia-associated plasmids are common
Source: PLoS Genet. 2022 Sep 19;18(9):e1010406. doi: 10.1371/journal.pgen.1010406 (PMC9560607; doi:10.1371/journal.pgen.1010406)

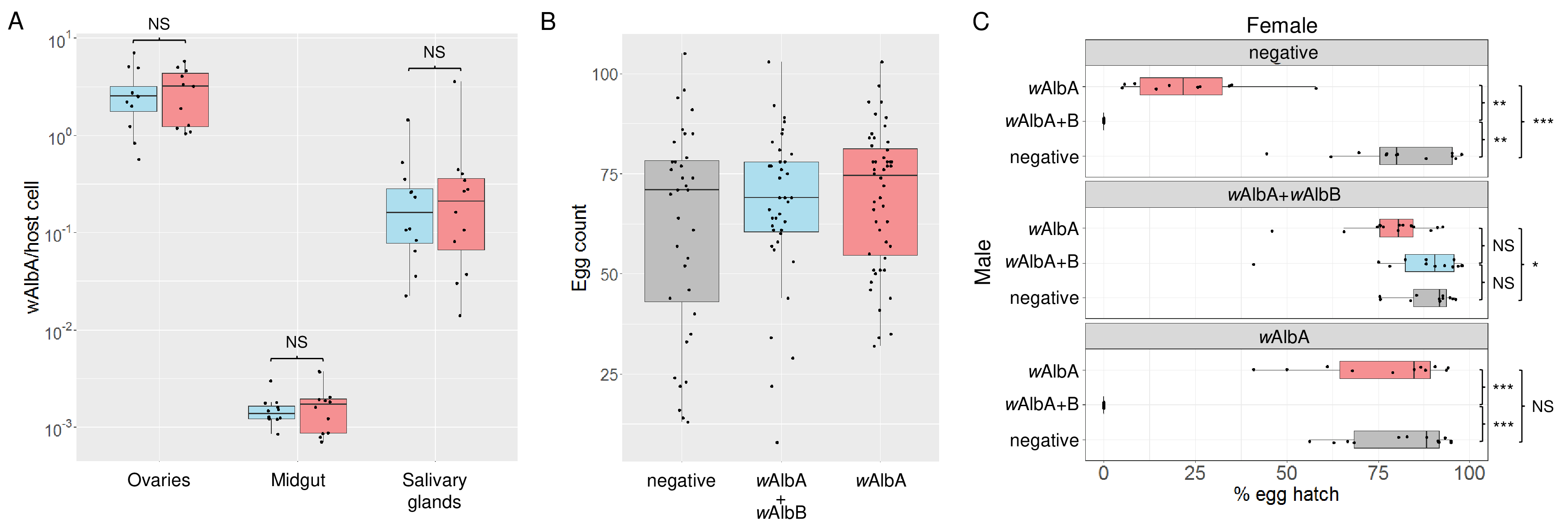

Supplement: S1 Fig — (A) wAlbA relative density within tissues of individual females. (B) Number of eggs laid by individual females. (C) Egg hatch rates in the progeny of individual females. Blue, red and grey indicate doubly-infected, wAlbA-infected and Wolbachia-free mosquito lines respectively. NS: non-significant; *: p < 0.05; **: p < 0.01; ***: p < 0.001. (JPEG) [file pgen.1010406.s001.jpeg]

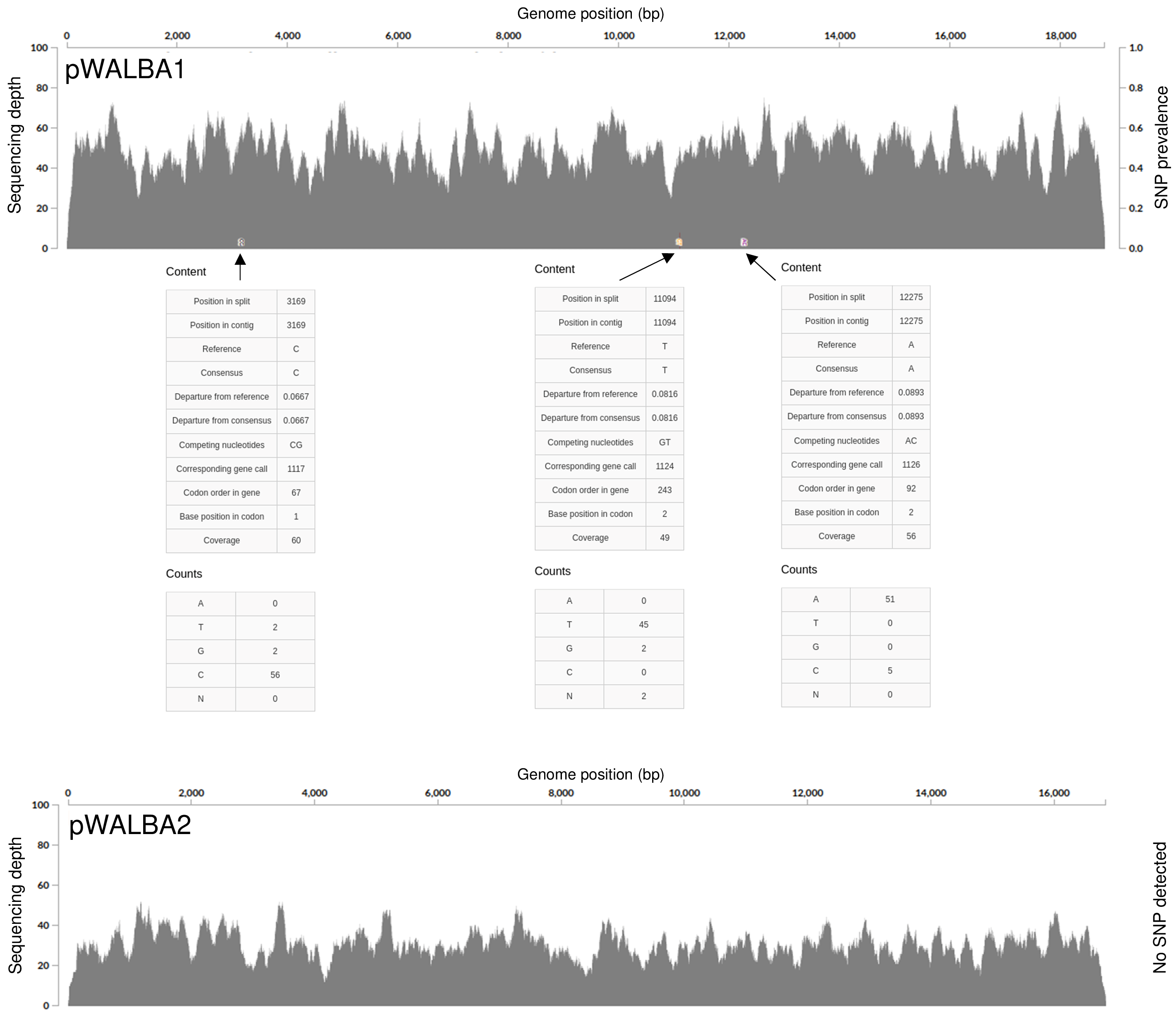

Supplement: S2 Fig — (TIF) [file pgen.1010406.s002.tif]

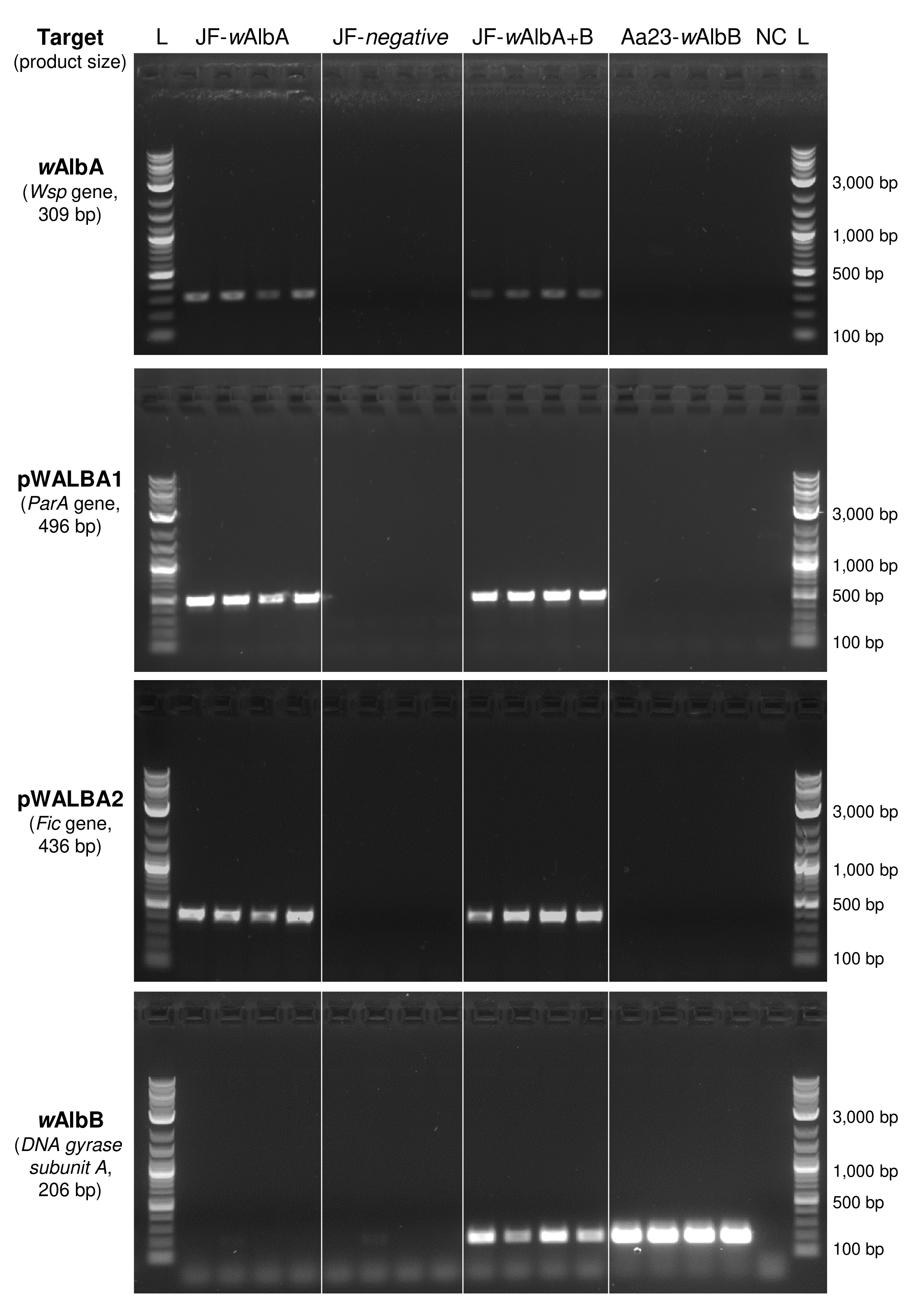

Supplement: S3 Fig — DNA was extracted from individual female mosquitoes and from the Aa23 cell line (n = 4 per Wolbachia infection status). Nomenclature: host background-Wolbachia infection status. NC: PCR negative control. L: DNA ladder. (JPEG) [file pgen.1010406.s003.jpeg]

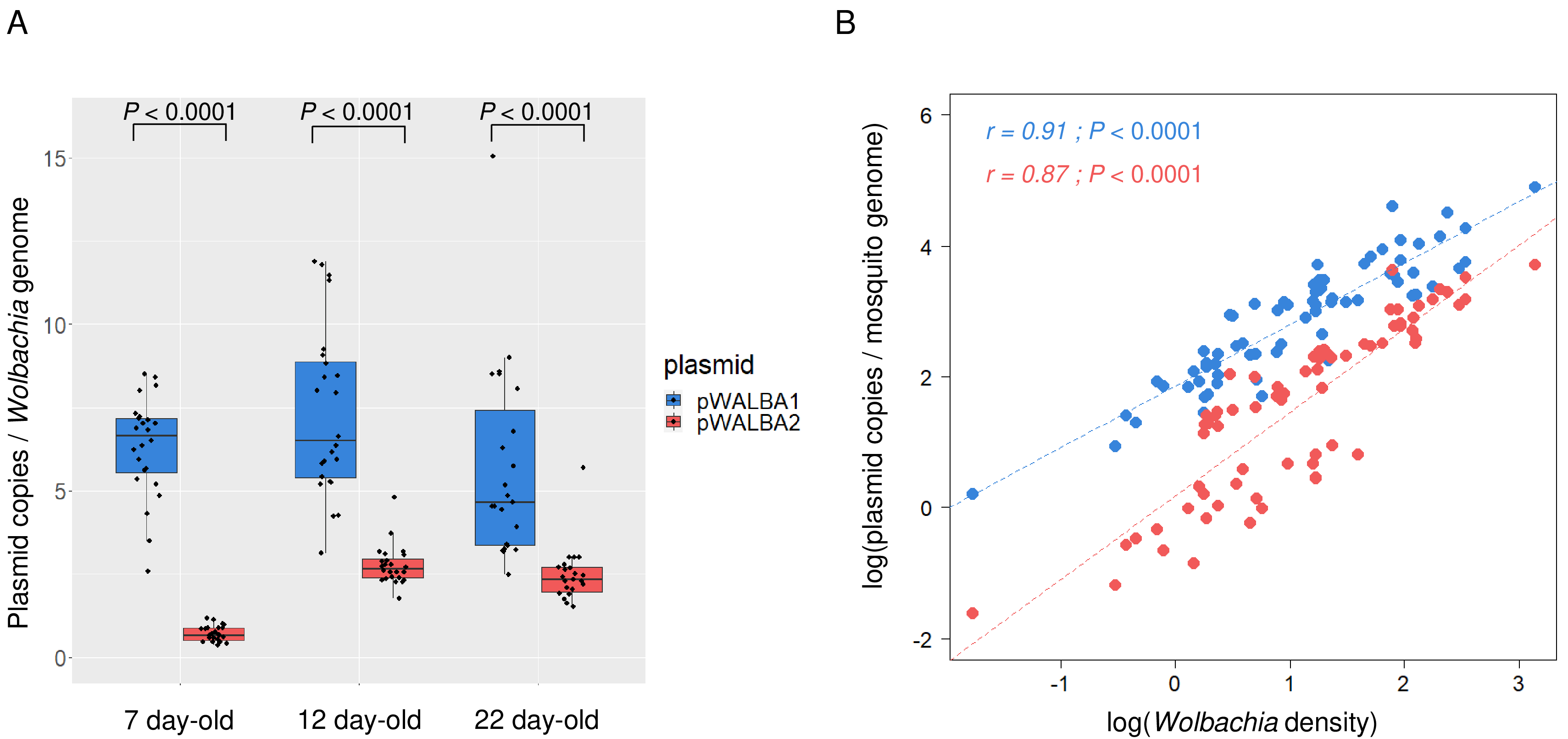

Supplement: S4 Fig — DNA was extracted from individual wAlbA-infected female mosquitoes at different timepoints. Females were blood-fed at 8 and 18 day-old. (A) Copy number of a plasmid-specific target (DnaB-like gene and Fic family protein for pWALBA1 and pWALBA2 respectively) relative to Wolbachia 16S rRNA copies. The p-values were calculated with a t test on paired log-transformed data. (B) Correlation between wAlbA densities and plasmid copy number per mosquito (blue: pWALBA1, red: pWALBA2). The dashed lines show predicted values from linear regressions and r is the Pearson’s correlation coefficient. (JPEG) [file pgen.1010406.s004.jpeg]

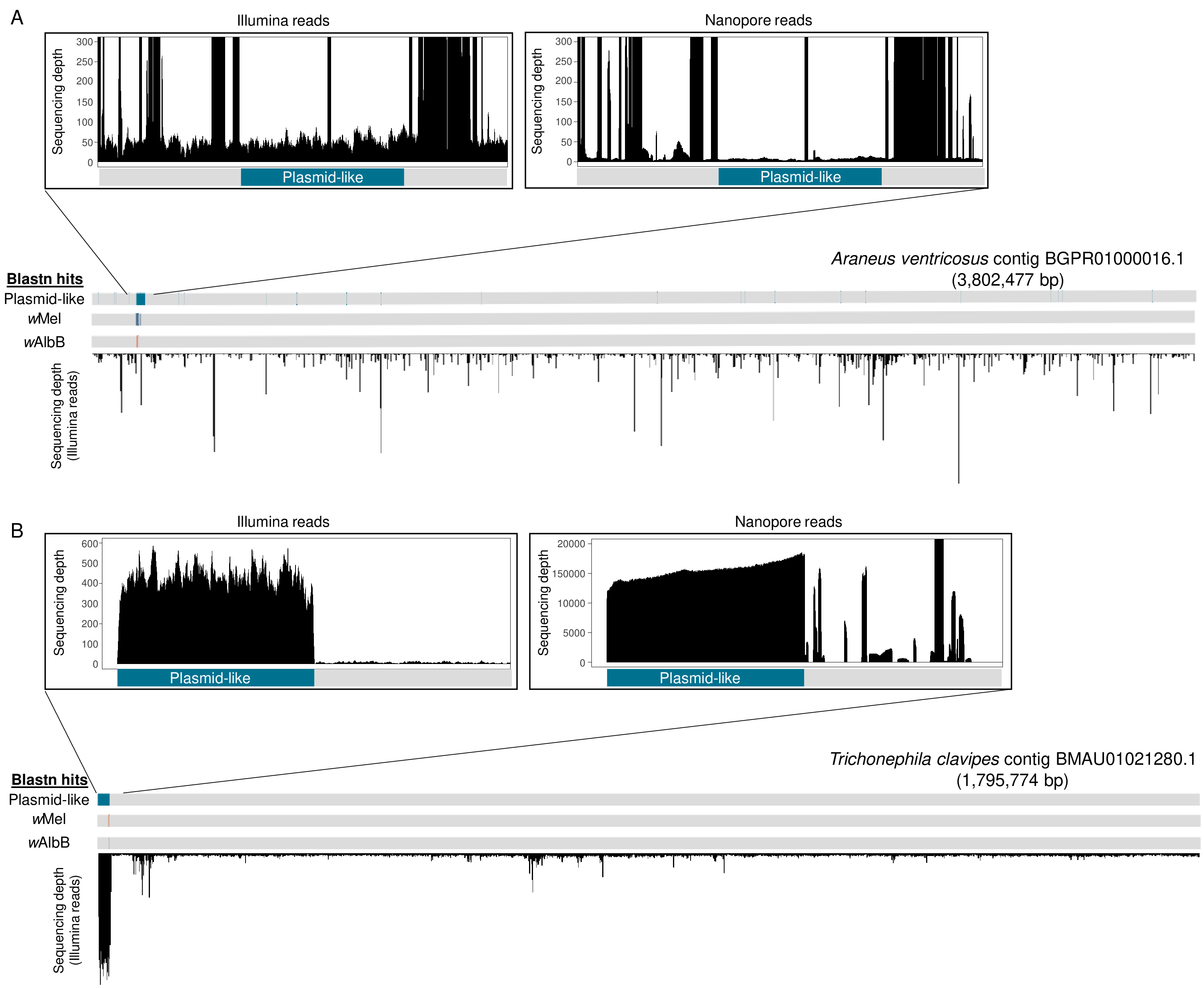

Supplement: S5 Fig — A. araneus (A) and T. clavipes (B) contigs were blasted against their respective plasmid-like region, the wMel (Supergroup A) and wAlbB (Supergroup B) reference genomes. BLASTN hits were visualized in Bandage. Sequencing depth was measured by mapping the Illumina and Nanopore reads from the corresponding sample onto the contig of interest. (JPEG) [file pgen.1010406.s005.jpeg]

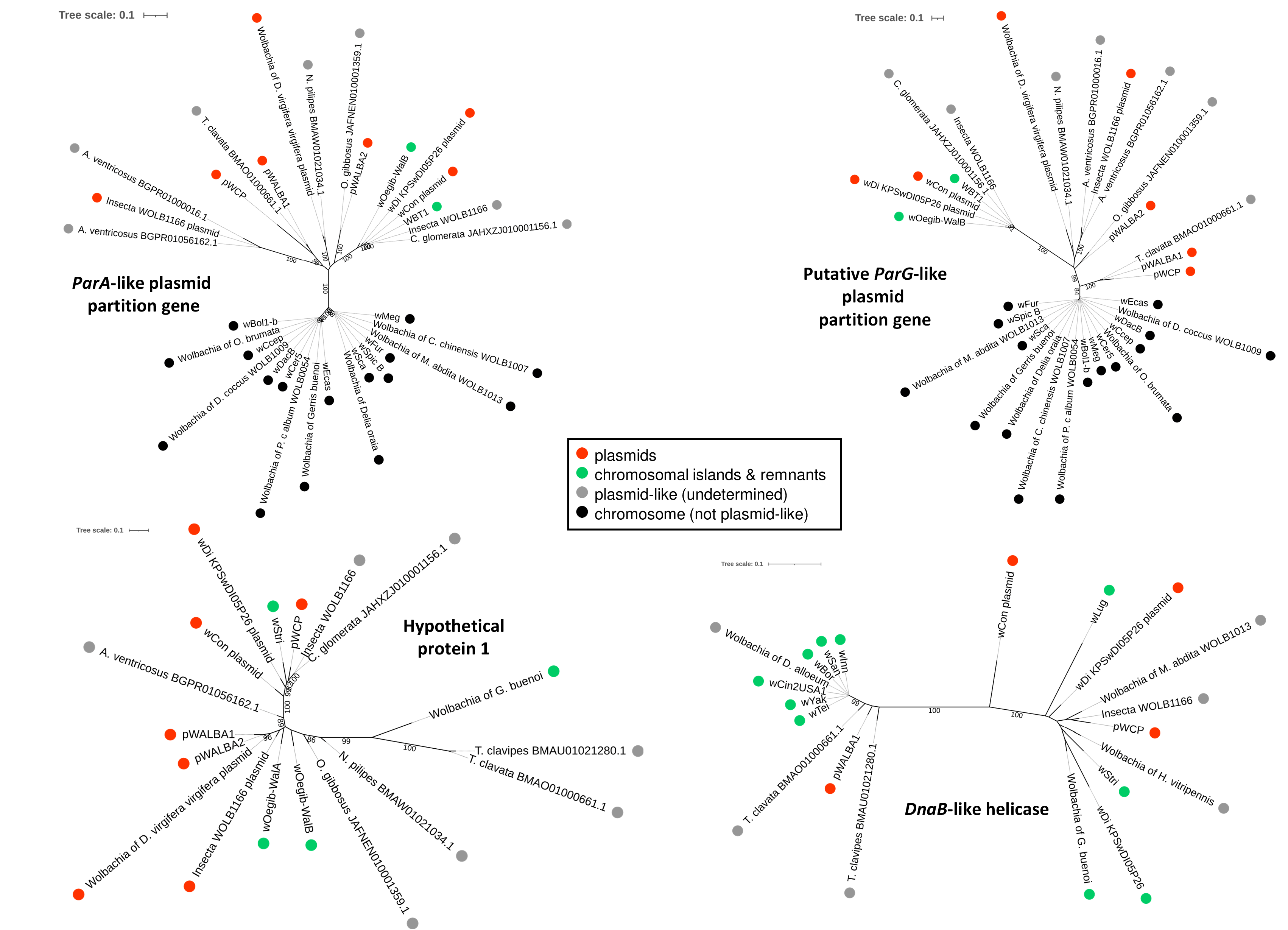

Supplement: S6 Fig — Full circles indicate the genomic location of the different homologues. Branch support calculated from 100 bootstraps replicates and >80% are shown. (JPEG) [file pgen.1010406.s006.jpeg]

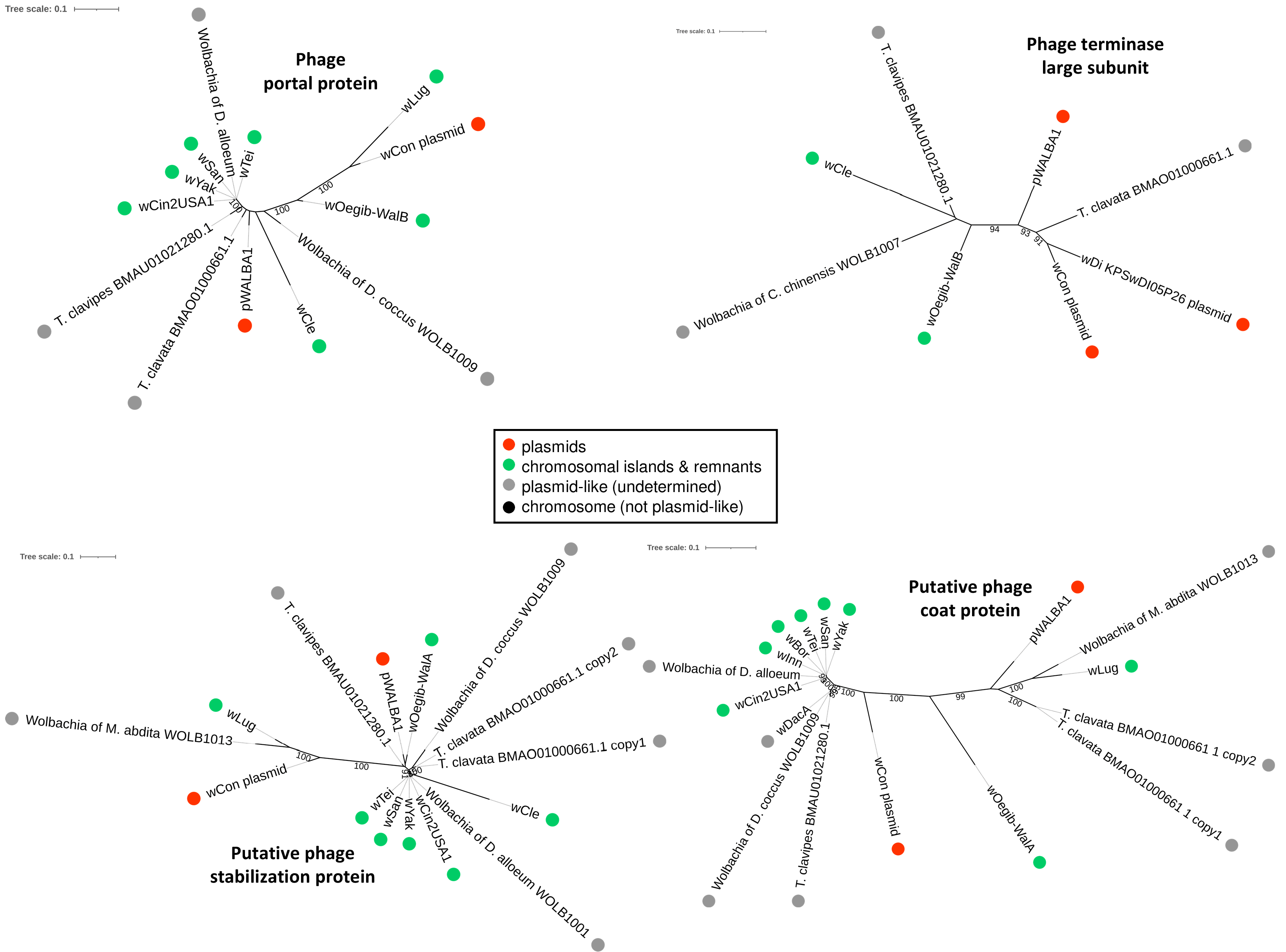

Supplement: S7 Fig — Full circles indicate the genomic location of the different homologues. Branch support calculated from 100 bootstraps replicates and >80% are shown. (JPEG) [file pgen.1010406.s007.jpeg]

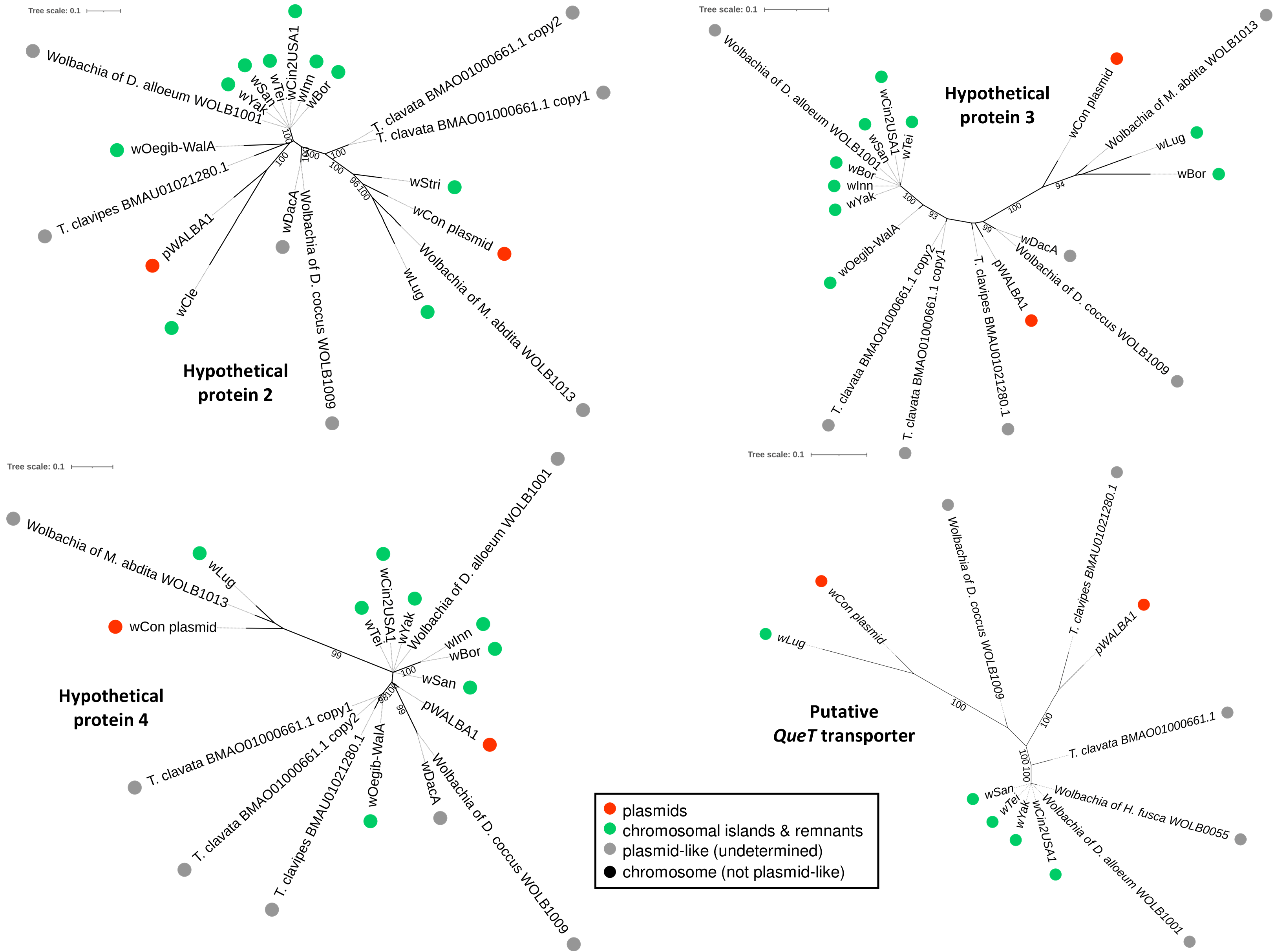

Supplement: S8 Fig — Full circles indicate the genomic location of the different homologues. Branch support calculated from 100 bootstraps replicates and >80% are shown. (JPEG) [file pgen.1010406.s008.jpeg]

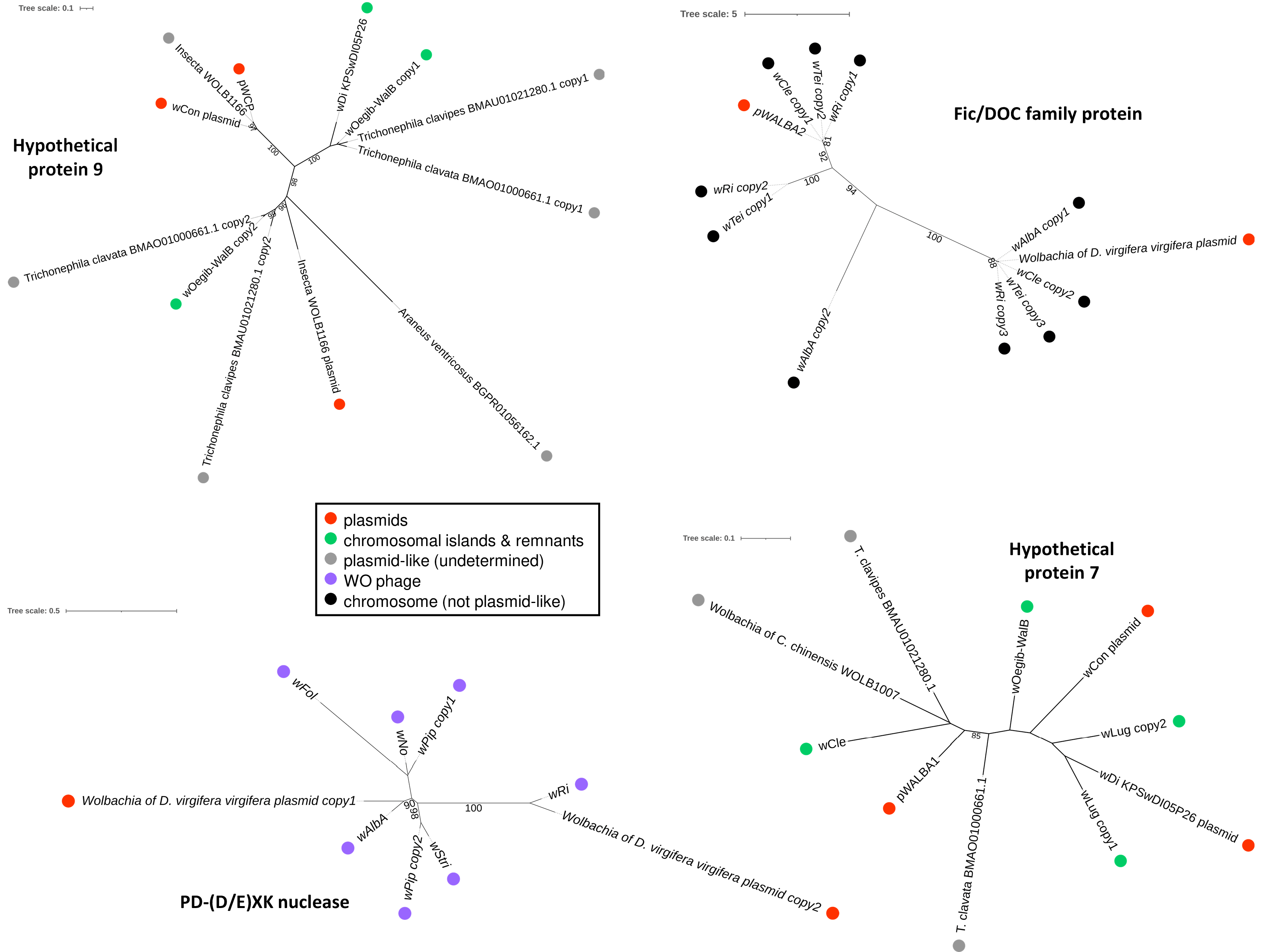

Supplement: S9 Fig — Full circles indicate the genomic location of the different homologues. Branch support calculated from 100 bootstraps replicates and >80% are shown. (JPEG) [file pgen.1010406.s009.jpeg]

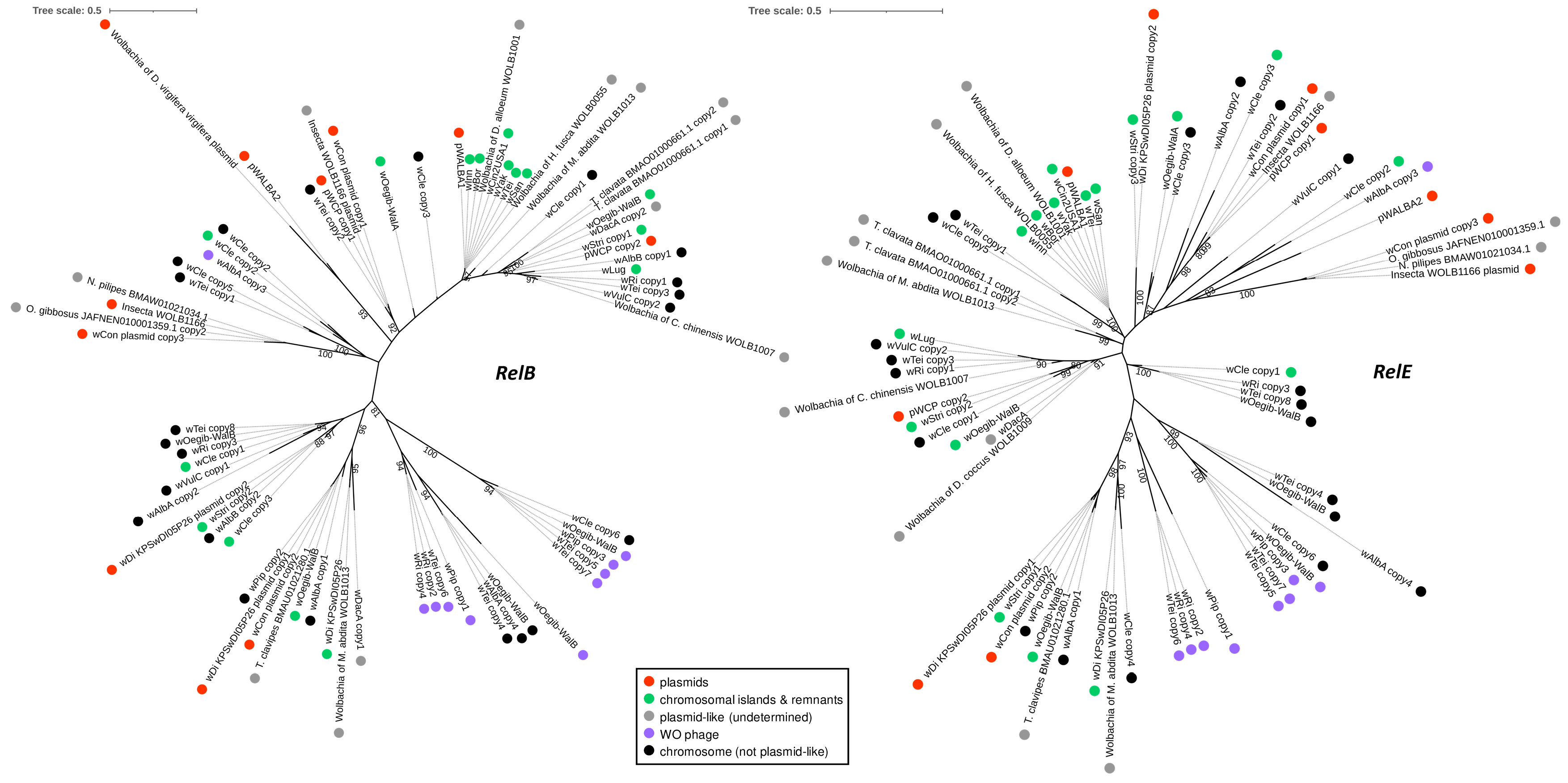

Supplement: S10 Fig — Full circles indicate the genomic location of the different homologues. Branch support calculated from 100 bootstraps replicates and >80% are shown. (JPEG) [file pgen.1010406.s010.jpeg]

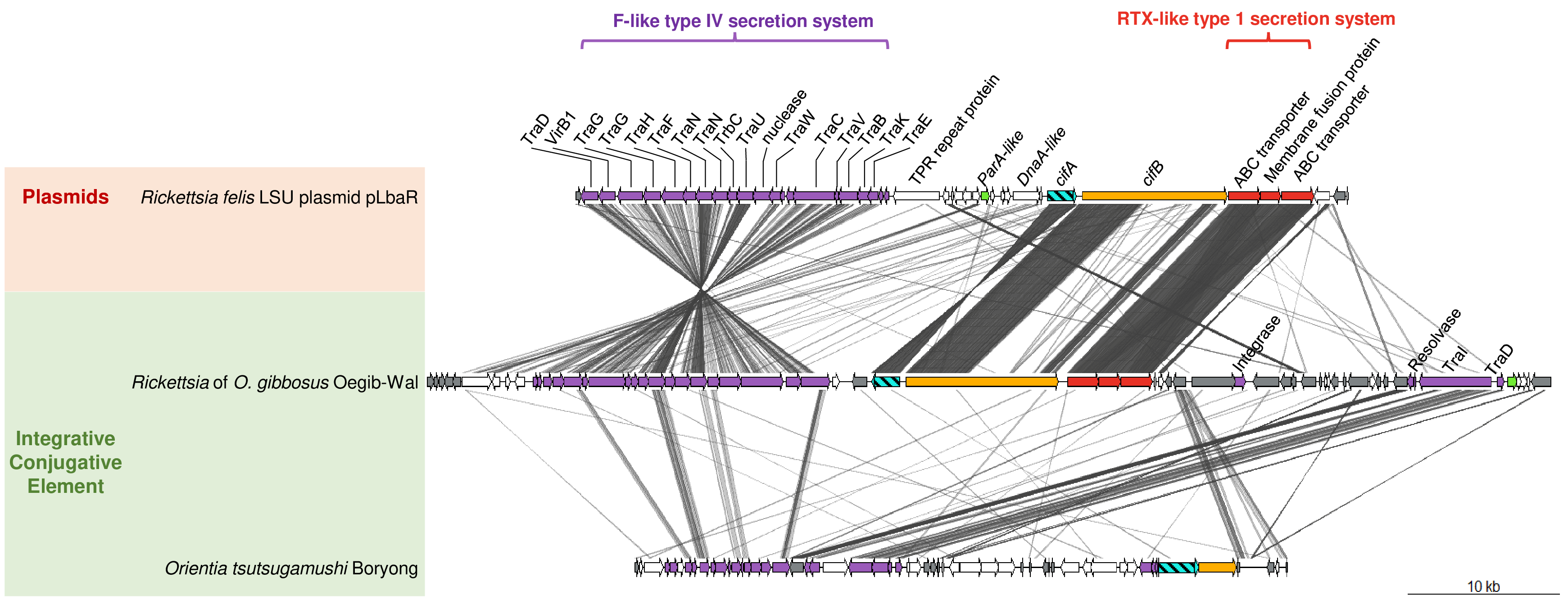

Supplement: S11 Fig — Similarity is indicated by gene colours (BLASTP) and by the grey areas between sequences (TBLASTX) where darker grey means more similar. Dark grey genes are transposable element sequences. (JPEG) [file pgen.1010406.s011.jpeg]
